# Behavioral and Gene Regulatory Responses to Developmental Drug Exposures in Zebrafish

**DOI:** 10.3389/fpsyt.2021.795175

**Published:** 2022-01-10

**Authors:** Aleksandra M. Mech, Munise Merteroglu, Ian M. Sealy, Muy-Teck Teh, Richard J. White, William Havelange, Caroline H. Brennan, Elisabeth M. Busch-Nentwich

**Affiliations:** ^1^School of Biological and Behavioural Sciences, Faculty of Science and Engineering, Queen Mary University of London, London, United Kingdom; ^2^Department of Medicine, Cambridge Institute of Therapeutic Immunology and Infectious Disease, University of Cambridge, Cambridge, United Kingdom; ^3^Centre for Oral Immunobiology and Regenerative Medicine, Institute of Dentistry, Barts and the London School of Medicine and Dentistry, Queen Mary University of London, England, United Kingdom

**Keywords:** developmental exposure, addiction, zebrafish, nicotine, oxycodone, amphetamine, behavior, RNA-seq

## Abstract

Developmental consequences of prenatal drug exposure have been reported in many human cohorts and animal studies. The long-lasting impact on the offspring—including motor and cognitive impairments, cranial and cardiac anomalies and increased prevalence of ADHD—is a socioeconomic burden worldwide. Identifying the molecular changes leading to developmental consequences could help ameliorate the deficits and limit the impact. In this study, we have used zebrafish, a well-established behavioral and genetic model with conserved drug response and reward pathways, to identify changes in behavior and cellular pathways in response to developmental exposure to amphetamine, nicotine or oxycodone. In the presence of the drug, exposed animals showed altered behavior, consistent with effects seen in mammalian systems, including impaired locomotion and altered habituation to acoustic startle. Differences in responses seen following acute and chronic exposure suggest adaptation to the presence of the drug. Transcriptomic analysis of exposed larvae revealed differential expression of numerous genes and alterations in many pathways, including those related to cell death, immunity and circadian rhythm regulation. Differential expression of circadian rhythm genes did not correlate with behavioral changes in the larvae, however, two of the circadian genes, *arntl2* and *per2*, were also differentially expressed at later stages of development, suggesting a long-lasting impact of developmental exposures on circadian gene expression. The immediate-early genes, *egr1, egr4, fosab*, and *junbb*, which are associated with synaptic plasticity, were downregulated by all three drugs and *in situ* hybridization showed that the expression for all four genes was reduced across all neuroanatomical regions, including brain regions implicated in reward processing, addiction and other psychiatric conditions. We anticipate that these early changes in gene expression in response to drug exposure are likely to contribute to the consequences of prenatal exposure and their discovery might pave the way to therapeutic intervention to ameliorate the long-lasting deficits.

## Introduction

Despite social awareness campaigns, drug usage amongst pregnant women in the USA remains high, standing at ~17% for nicotine, ~8.5% for alcohol and 5.9% for illicit drugs such as cocaine, methamphetamine, marijuana and prescription-type psychotherapeutics (1). Prenatal drug exposure poses a significant health risk for the developing fetus, either directly by crossing the placenta and acting on molecular targets in the fetus, indirectly through physiological effects on the mother, or a combination of both. The most common effects seen in newborns that have been exposed to drugs of abuse during gestation include growth restriction, decreased weight and cranial and cardiac anomalies (2, 3). However, prenatal drug exposure is also associated with increased vulnerability to psychiatric disease, including addiction (4), schizophrenia (5), autism (6) and ADHD (7), as well as aggression, peer-related problems and learning difficulties (8–11). These findings suggest that drug exposures at developmental stages lead to profound changes that last beyond the exposure period, manifesting both as motor and cognitive impairments and as phenotypes associated with addiction and other psychiatric disorders.

Although the consequences of developmental exposure to drugs of abuse in terms of neural development are not fully understood, a number of studies have shown altered expression of key components of neurotransmitter pathways in regions of the brain associated with behavioral responses and long-term changes in behavior. For example, prenatal methamphetamine exposure in rats showed, among other changes, altered expression of dopamine receptors (Drd3) in the striatum in adulthood (12). Reduced release of dopamine was reported in adult mice following prenatal nicotine exposure (13). Additionally, nicotine exposure has been linked to neuronal loss in striatal and hippocampal regions in adult rats, both of which play a critical role in learning and memory (14, 15). Similarly, alteration in these regions were observed following *in-utero* opioid exposure in humans (16). Widespread neuroapoptosis throughout the developing brain of several species, mechanisms of which are not fully understood, is also reported following prenatal drug exposures (17). Recent evidence from rodent studies suggests that prenatal and postnatal drug exposures lead to changes in gene expression as a result of altered DNA methylation (18, 19). More details on the effect of developmental exposure on development can be found in recent reviews: (20–22).

In this study, we have used zebrafish to investigate the changes in gene expression following developmental exposure to three commonly abused drugs to gain insight into alterations in biological pathways that may contribute to changes in behavior in later life. The zebrafish, a well-established behavioral and genetic model with conserved drug response and reward pathways (23, 24), has rapid *ex utero* embryogenesis which allows non-invasive drug treatments at early embryonic stages. We exposed developing zebrafish to three drugs that are commonly abused by women of reproductive age: amphetamine, oxycodone and nicotine (25). We have chosen drugs with different modes of action—two stimulants, amphetamine and nicotine, and an opioid, oxycodone—in order to investigate both overlapping and drug class specific changes in gene expression.

Amphetamine is a dopamine (DA) transporter (DAT) inhibitor that prevents presynaptic reuptake of DA and therefore increases the concentration of dopamine and noradrenaline at the synapse, leading to a psychostimulant response (26). Nicotine is a strong alkaloid whose main mechanism of action in the human body is through binding to nicotinic acetylcholine receptors (nAChRs). Consumed nicotine stimulates nAChRs in the central nervous system (CNS) which, in the developed brain, causes a release of dopamine and also glutamate, serotonin, acetylcholine and γ-aminobutyric acid (GABA) (27).

Oxycodone acts by attaching to μ-opioid receptors on the surface of neurons. In adults, activation of the μ-opioid receptors sends a signal through the ventral tegmental area (VTA) that causes the release of dopamine in the NAc and gives a feeling of pleasure to the user (28). Oxycodone is widely used as a pain relief medication in post-operative, chronic and cancer-related pain management (29). Despite having many beneficial effects it is also one of the most addictive prescription drugs on the market. Currently, the USA has an opioid epidemic with overdose death rates increasing at alarming rates. Therefore, understanding the potential consequences of developmental oxycodone exposure is crucial to ameliorating the impact of this crisis on future generations (30).

We exposed developing zebrafish to amphetamine, nicotine or oxycodone from 1 to 5 days post fertilization (dpf), the period during which all major organ systems develop and begin functioning (31). In the presence of drugs, developmentally exposed larvae showed changes in locomotion and habituation to acoustic startle, consistent with the effects of these drugs in mammalian systems. We show that developmental exposure induces differential expression of numerous genes and alterations in many pathways, including those involved in development, cell death regulation, circadian rhythm, innate immunity and synaptic plasticity.

## Materials and Methods

### Zebrafish Husbandry

All *in vivo* experimental work was carried out following consultation of the ARRIVE guidelines (NC3Rs, UK). Zebrafish were maintained in accordance with UK Home Office regulations, UK Animals (Scientific Procedures) Act 1986. All animal work was reviewed by the Animal Welfare and Ethical Review Body at the University of Cambridge (project license P597E5E82) and by the Animal Care and Use ethics committee at Queen Mary University of London (project license P6D11FBCD).

Fish were housed in a recirculating system (Tecniplast, UK) on a 14:10 light:dark cycle. The housing and testing rooms were maintained at ~25–28°C. Fish were maintained in aquarium-treated water and fed twice daily with flake food (ZM-400, Zebrafish Management Ltd, Winchester, United Kingdom) and once daily with live artemia (*Artemia salina*). All zebrafish used in this study originated from a Tupfel long fin (TLF) wild-type (WT) background line. All animals were selected at random from groups of conspecifics for testing.

At the time of mating, breeding males and females were separated overnight before letting them spawn naturally in the morning to allow for synchronization of developmental stages. Eggs were incubated in groups of no more than 50 per Petri dish at 28°C until 5 days post fertilization (dpf). Then, larvae were transferred to the recirculating system and fed twice daily with commercial fry food (ZM-75, ZM-100, Zebrafish Management Ltd, Winchester, United Kingdom) and live paramecia/brine shrimp, depending on their age.

### Drug Exposure

For developmental drug exposure, wild-type TLF strain prim-5 stage embryos (24 h post fertilization) were divided into three treatment groups and a control group and exposed until 5 dpf. Drug solutions were prepared in egg water to final concentrations of 25 μM amphetamine (Sigma-Aldrich, Cat. No. A5880), 5 μM nicotine (Sigma-Aldrich, Cat. No. N1019) and 1.14 μM oxycodone (Sigma-Aldrich, Cat. No. O1378). Drug concentrations were based on those found to induce maximal conditioned place preference in adults (32). We aimed to model consistent human consumption during pregnancy. The concentrations present in the fish water were higher than those reported to be present in the human fetal blood during pregnancy (33, 34). However, as the pharmacokinetics of the compounds is not known in fish, we selected concentrations that have been previously shown to have a relevant physiological effect in zebrafish when administered via the tank water. Even though the magnitude of any changes in gene expression may differ, we predict that the direction of the changes remains the same. Although oxycodone and amphetamine are stable in water for at least 24 h (35, 36), there is contrasting evidence for the rate of decline in nicotine concentration over time with some evidence suggesting a rapid decline over a 48-h period (personal communication, Sala) and others pointing at decline over a 10 day period (37). To account for potential degradation and for consistency in handling across treatments, we therefore refreshed all solutions every 48 h. At 5 dpf, either solutions were refreshed and larvae taken for behavioral or RNAseq analysis, or larvae were transferred to fresh Petri dishes in the absence of drug and reared.

For acute drug exposure, wild-type TLF larvae were raised in egg water until 5 dpf. An hour before the start of the behavioral experiments water was removed and replaced with freshly made drug solutions of 25 μM amphetamine, 5 μM nicotine and 1.14 μM oxycodone.

### Behavioral Assays

To ensure drug penetration we examined locomotor activity in the presence of the drug and post exposure. We performed two independent larval behavioral assays: forced light-dark assay (FLD) and habituation to acoustic startle. FLD is commonly used as an anxiety measure, based on the assumption that a sudden change of environment from dark to light will increase locomotion through the activation of the hypothalamic-pituitary-interrenal (HPI) axis (38). Habituation to acoustic startle is a measure of sensorimotor gating, a process that is modulated by dopaminergic signaling (39).

Eggs for all behavioral experiments were collected from multiple adult pairs, pooled and divided across at least three Petri-dishes. Fertile eggs were staged-matched and randomly assigned to treatment groups. Embryos and larvae were carefully monitored for differences in development across dishes. For all experiments, stage-matched larvae were selected from all Petri-dishes, to account for possible batch/dish effects. No morphological or immediate behavioral differences were seen between treatment and control groups in the 5 dpf larvae following developmental drug exposure prior to behavioral analysis. All tested individuals were the same size. Larvae were culled after behavioral experimentation.

#### Forced Light-Dark Transition

Experiments were performed between 1 and 6 p.m. Behavior was assessed at 5 dpf in the presence of the drug, or at 6 dpf, in the absence of the drug, 24 h after the end of the exposure. Larvae were individually placed in 96-well plates, pseudo-randomized by drug treatment, and allowed to acclimate for 30 min. After this period, the plate was placed inside a DanioVision Observational Chamber (Noldus Information Technology, Wageningen, The Netherlands). Larval locomotion was recorded during alternating light/dark periods: 10 min in the dark (infrared conditions), which was used as a baseline; 10 min in the light; 10 min in the dark; 1 min in the light; 10 min in the dark. Distance traveled was recorded using Ethovision XT software (Noldus Information Technology, Wageningen, The Netherlands) and data were output in 10 and 1 s time bins for analysis. Larvae were culled after behavioral experimentation.

#### Acoustic Startle Habituation

Acoustic startle was performed as described previously (40) with minor modifications. Experiments were performed between 1 and 6 p.m. Behavior was assessed at 5 dpf in the presence of the drug, or at 6 dpf, in the absence of the drug, 24 h after the end of the exposure. Larvae were individually placed in 96-well plates, pseudo-randomized by drug treatment, and allowed to acclimate for 30 min. After this period, the plate was moved to a DanioVision Observational Chamber (Noldus Information Technology, Wageningen, The Netherlands) where, following a 2-min acclimatization period, larval movement was recorded. Following an initial 1 min baseline period, larvae were subjected to 25 sound/vibration stimuli with 2 s inter-stimulus intervals. The distance traveled was recorded using Ethovision XT software (Noldus Information Technology, Wageningen, The Netherlands), and data were output in 1 s time bins.

#### Exploratory/Bold Behavior Assay

The exploratory/bold behavior assay was a modified version of a sociability assay performed by Dreosti et al. (41). All experiments were performed between 12 and 7 p.m. with age- and size-matched subject and stimulus fish. Briefly, fish were reared in groups of 50 until 20–22 dpf, a period that can be considered as corresponding to adolescence (puberty) in humans due to the intense growth and transition from sexual immaturity to maturity that zebrafish undergo within 9–51 dpf (42). Individual fish were then placed in the center of a U-shaped choice chamber. The final third of each arm of the U-shape was separated from the rest of the apparatus by a glass partition. Fish were allowed to explore the apparatus for 30 min. During the next 15 min, three conspecific fish were added to one of the partitioned areas and the time the test fish spent on each side of the apparatus was recorded. Twelve individual fish were tested simultaneously in two DanioVision Observation Chambers (Noldus Information Technology, Wageningen, The Netherlands). Swimming activity and position within the arena were recorded using Ethovision XT software (Noldus Information Technology, Wageningen, The Netherlands) and the data were output in 15-min time bins. To assess social behavior two measures were calculated: Social Preference Index (SPI), as previously described (41), and Correlation Index (r), which assesses fish predisposition to socialize: [*r* = SPIExperimentalPhase – SPIAcclimationPeriod]. The Correlation Index was also used as a measure of exploratory/bold behavior—bold fish spend more time away from stimuli and therefore values of their Correlation Index are positive. The percentage of individuals with a positive Correlation Index is interpreted as the percentage of fish displaying exploratory/bold behavior.

#### Circadian Rhythm

Developmentally exposed larvae were raised in a dark incubator without a 14:10 light:dark cycle. Following the end of exposure, 5 dpf larvae in the absence of the drug were individually placed in a 96-well plate, pseudo-randomized by treatment, and left in the light for 5 h. The plate was then moved to a DanioVision Observational Chamber (Noldus Information Technology, Wageningen, The Netherlands) where fish movement was recorded over 59 h under different light conditions: 3 h light; 54 h dark; 2 h light. Distance traveled by larvae during the assay was recorded using Ethovision XT software (Noldus Information Technology, Wageningen, The Netherlands) and the data were output in 1-min time bins for analysis. Periodicity was assessed using the R package DiscoRhythm (43).

#### Response to Dusk/Dawn

Developmentally exposed larvae were raised in a dark incubator without a 14:10 light:dark cycle. Following the end of exposure, 5 dpf larvae in the absence of the drug were individually placed in a 96-well plate, pseudo-randomized by treatment, and allowed to acclimate in the light for 30 min. The plate was then moved to a DanioVision Observational Chamber (Noldus Information Technology, Wageningen, The Netherlands) where fish movement was recorded over 16.5 h under different light conditions: 3 h light; 46 min gradual dusk; 10 h dark; 46 min gradual dawn; 2 h light. During gradual dusk, light intensity was decreased by 5% every 2 min until 5% intensity was reached and then every 2 min by 1% until the light went off. During gradual dawn, light intensity was increased every 2 min by 1% until 5% intensity was reached and then by 5% until 100% intensity was reached. Distance traveled by larvae during the assay was recorded using Ethovision XT software (Noldus Information Technology, Wageningen, The Netherlands) and the data were output in 1-min time bins for analysis.

#### Data Analysis

For analysis and visualization of behavioral data R version 4.0.5 (44) and RStudio version 1.4.1106 (45) were used. Data analysis was performed as previously described (40, 46) with slight changes. For FLD, four subsets of the data were created and analyzed separately: baseline, light and dark periods and startle response. All the periods were fitted to a linear mixed model with the mean distance traveled as a response variable, condition as a fixed effect and fish ID as a random effect. During alternating light and dark periods larvae movement increases in light over time. To explore this behavior, linear models of light periods were fitted using distance traveled as a response variable, the interaction between condition and time as an independent variable and fish ID as a random effect. The β coefficient in light period models represents the increase in distance traveled over time and can be interpreted as the larval “recovery rate.” To explore anxiety-related responses to light, startle response was calculated as the distance moved during the first 20 s following light transition divided by the mean distance moved during the 1-min light event. The duration of the startle response was taken as 20 s following the transition from light to dark, as after this time all conditions started to recover from the initial startle response. Distance moved during the 1-min light period and startle response was calculated separately for each fish to account for differences in locomotion. This proportion was analyzed using the R package “betareg” (47), with the proportion of startle response as a response variable and condition as an explanatory variable.

For the response to acoustic startle, the data were divided into two parts—baseline period and response to startle stimuli. The baseline period was analyzed as described above. The response to startle stimuli was analyzed by implementing two approaches. For both, each stimulus (tap) event was defined as a two second event. In the first approach, the slope of habituation to startle stimuli was calculated by fitting a linear mixed model using distance traveled as a response variable, the interaction between condition and tap event as an independent variable and fish ID as a random effect. Then, significant fixed effects were identified using a chi-squared test and, when significant differences were established, *post-hoc* Tukey tests were used to further characterize the effects. In the second approach, a response/non-response status was defined for each fish. The threshold for response status was defined as the mean distance moved per second during the basal period plus two standard deviations (SD) of the mean. As there were significant differences in basal locomotion, thresholds were calculated separately for each condition. For each tap event, each fish was assigned as being a “responder” if it moved more than the threshold during the tap event or as a “non-responder” if it did not. Beta regression was modeled with the percentage of fish responding to a stimulus as a response variable and the interaction between tap event number and condition as an explanatory variable. Then, likelihood ratio tests for nested regression models were performed to assess if the interaction between the tap event number and condition was a significant predictor of individual responsiveness.

Linear mixed models were calculated using the R package lme4 (48) and significant fixed effects were identified using a chi-squared test. To further characterize the effects, where significant differences were established, *post hoc* Tukey tests were conducted using the R package “multcomp” (49).

### RNA-Seq Sample Collection and Preparation

At the end of the exposure period (1–5 dpf), larvae were collected as six pools of seven embryos per condition for RNA extraction to minimize any differences due to biological variance. A previously described protocol for single embryo RNA extraction (50) was optimized for use with pools of zebrafish larvae. Samples were lysed in 110 μl RLT buffer (Qiagen) containing 1.1 μl of 14.3 M β-mercaptoethanol (Sigma). The lysate was allowed to bind to 450 μl of Agencourt AMPure XP beads (Beckman Coulter) for 15 min. The tubes were left on a magnet (Invitrogen) until the solutions cleared and the supernatant was removed without disturbing the beads. Whilst still on the magnet, beads were washed three times with 70% ethanol and allowed to dry for 20 min. Total nucleic acid was eluted from the beads following the manufacturer's instructions and treated with DNase I (NEB, Catalog number M0303L). RNA was quantified using a NanoDrop (Thermo Scientific NanoDrop One Microvolume UV-Vis Spectrophotometer), RNA integrity numbers were checked using a Bioanalyzer (2100 Bioanalyzer System) and sequencing libraries were made using the Illumina TruSeq Stranded mRNA Sample Prep Kit.

### RNA Sequencing and Analysis

Libraries were pooled and sequenced on one lane of NovaSeq SP PE50 in 54 Gbp single-end mode (between 16 and 24 million reads per sample). RNA-seq data have been deposited in the ArrayExpress database at EMBL-EBI (www.ebi.ac.uk/arrayexpress) under accession number E-MTAB-11086. Sequencing data were assessed using FastQC and aligned to the GRCz11 reference genome using STAR (51). Two samples were excluded from data analysis after QC and visual inspection of a Principal Component Analysis: one control sample had a poor RNA integrity number and was degraded and one nicotine sample did not cluster with the rest of the samples.

Read counts per gene were generated by STAR and used as input for differential expression analysis using the R package DESeq2 (52). The following model was used for DESeq2: ~Treatment, modeling counts as a function of the drug treatment. Genes with an adjusted *p*-value of <0.05 were considered to be differentially expressed. Gene sets were analyzed for GO enrichment using topGO.

For analysis of gene expression changes and visualization of data, R version 4.0.5 (44) and RStudio version 1.4.1106 (45) were used. The following packages were utilized: tidyverse (53) for data manipulation; DESeq2 (52), ggfortify (54) and ggplot2 (55) for principal component analysis (PCA); GOPlot (56) and VennDiagram (57) for analysis of overlapping gene expression changes; pheatmap (58) for generating heatmaps; ggplot2 and ggrepel (59) for other plots, including the volcano plots.

### Whole-Mount mRNA *in situ* Hybridization

DIG-labeled RNA probes were generated from cDNA libraries (SuperScriptR IV Reverse Transcriptase, Invitrogen) covering stages of development where genes of interest are expressed. PCR was performed with site-specific primers containing a flanking T7 promoter sequence to produce DNA templates. Then, DIG-labeled RNA probes were generated by enabling *in vitro* transcription using T7 RNA polymerase (Roche). Oligonucleotide sequences for the primers can be found in Supplementary Table 1.

Larvae were fixed in 4% PFA (in PBS) at 4°C overnight and progressively dehydrated in ascending concentrations of ethanol (25, 50, 75, and 100%) before being kept in 100% methanol at −20°C until needed. We optimized previously described ISH protocols in zebrafish (60, 61) for 5 dpf larvae and for transcripts of interest. Larvae were incubated for 1 h at room temperature in ethanol/xylol solution (1:1 vol/vol) and then progressively rehydrated in descending concentrations of ethanol (90, 75, 50, 25% and then H_2_O). Eighty percentage acetone treatment at −20°C for 30 min was used for permeabilization followed by bleaching for 1 h in 6% H_2_O_2_. Pre-hybridization was performed at 65°C for 4 h, followed by extended hybridization of approximately 60 h in 50–100 ng/ml of probe at 65°C. Following hybridization, washed larvae were incubated in blocking solution (10% sheep serum diluted in TBST, TBS containing 0.1% Tween20) for 4 h at room temperature and then in alkaline phosphatase anti-DIG antibody (1:2,000) at 4°C overnight.

To remove non-specifically bound antibodies, extended periods of washings (two overnights in TBST) were performed and the larvae were stained using 1 ml of BM Purple. The reaction was stopped when the desired intensity was reached by washing the larvae in PBST (PBS containing 0.1% Tween20) and then in ascending concentrations of ethanol (25, 50, and 70%) to increase contrast. A camera set up on a dissecting microscope (Leica MZ9.5 Stereozoom) with a white background and white-light illumination from the top was used for imaging.

### Quantitative Real Time PCR

Following the end of drug exposure (1–5 dpf), larvae were raised in freshwater in the absence of a drug until 7 or 21 dpf. Samples for qPCR analysis were collected as 10 pools of 5 larvae (at 7 dpf), or 10 pools of 3 heads (at 21 dpf) for each treatment. Samples were kept in RNAlater (Thermo Scientific) until use. RNA was extracted using TRIzol reagent following the protocol provided by the manufacturer and described previously (62). RNA yield and quality were determined using a Thermo NanoDrop 2000 (ThermoFisher). Following the treatment of RNA extracts with DNase I (New England Biolabs), the cDNA libraries were created using the ProtoScript II First Strand cDNA Synthesis Kit (New England Biolabs) as suggested by the manufacturer and described previously (62). Gene expression levels were quantified using the LightCycler 480 qPCR system (Roche) based on our previously published MIQE-compliant (63) protocols (64, 65). mRNA expression levels were checked for six circadian rhythm associated genes that were differentially expressed in RNA-seq analysis: *cry1a, per2, per1a, cry3b, cry5*, and *arntl2*. Reference housekeeping genes β*-actin* and *18s* were chosen based on previous studies (66). Primer sequences for the genes can be found in Supplementary Table 2.

## Results

### Forced Light/Dark Transition: Drug Exposure Affects Larval Locomotion

First, we assessed locomotion and anxiety-like behavior in control- and drug-exposed larvae using a forced light-dark (FLD) transition assay consisting of 10 min in the dark (baseline period) followed by 10 min light and 10 min in the dark. At 5 dpf in the presence of the drug, exposure to 25 μM amphetamine and 5 μM nicotine resulted in significant changes in basal locomotion when compared to controls (*p* < 0.001; *p* = 0.0396, respectively) such that treated fish moved less. No significant changes were observed for oxycodone-exposed fish in the baseline period. These changes in locomotion were prevalent for amphetamine- and nicotine-treated fish throughout the duration of the assay (light period: *p* < 0.001; *p* < 0.001; dark period: *p* < 0.001; *p* = 0.0031, respectively). Additionally, oxycodone-treated fish moved less than controls during the 10 min light period (*p* = 0.0033).

In response to the transition from dark to light, unexposed control fish displayed an initial period of freezing/reduced movement that gradually increased toward baseline levels over the 10 min period in the light. We assessed these changes in locomotion during the light period measured as the slope from min 10 to 20. There were significant differences in the rate of recovery during the light period, where nicotine-treated fish showed slower recovery than untreated fish (*F* = 6.70e-06, *p* = 0.0002).

**Figure 1:**
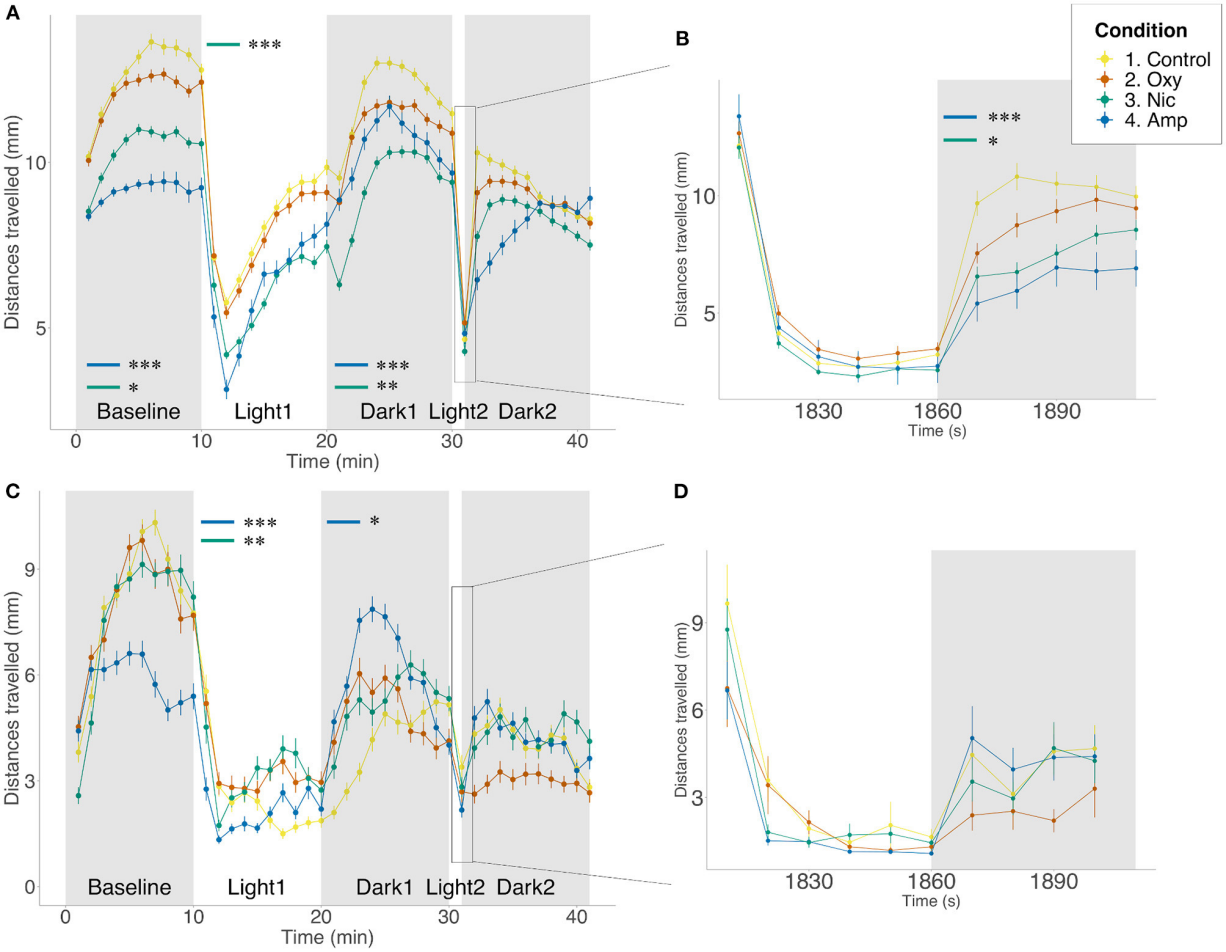
Differences in locomotion in FLD assay. Mean distance traveled per minute by larvae from each treatment group during alternating dark (gray) and light (white) periods. Startle response following 1 min light exposure is displayed in a 10-s resolution plot **(B,D)**. **(A,B)** Light/dark-induced locomotor responses in 5 dpf larvae in the presence of drug. Sample size *n* = 96 for each group. **(C,D)** Light/dark-induced locomotor responses in 6 dpf larvae in the absence of drug. Sample size *n* = 48 for each group. Significant differences are indicated where present: for locomotion during dark periods (minute 0–10 and 20–30); for slope of recovery in light periods (minute 10–20) and for startle response following 1 min light exposure **(B,D)**. Data shows mean ± SEM. Data was fitted to the linear mixed effect model and *post-hoc* Tukey test was used to identify multiple correlations (*p < 0.05, **p < 0.01, ***p < 0.001 Tukey's multiple comparison test).

We also assessed anxiety-like behavior in response to a short 1 min exposure to light followed by 5 min in the dark. Here, on transition from light to dark, unexposed fish exhibited an initial startle response seen as a rapid increase in movement that gradually decreased in darkness. The startle response of both amphetamine- and nicotine-treated fish was significantly smaller than controls (*p* = 0.0004; *p* = 0.0190, respectively). We assessed movement and slope of recovery during the 5 min dark period following 1 min light exposure, in accordance with previously published protocols (38). There was a significant difference in distance moved, where amphetamine-treated fish moved less than controls (*p* < 0.001). Although oxycodone-treated fish showed reduced locomotion, this did not reach significance (*p* = 0.0697). We observed differences in the rate of recovery during this time, such that all treated fish recovered more quickly than controls (amphetamine-treated: *F* = −2.96e-05, *p* < 0.0001; nicotine-treated: *F* = −2.00e-05, *p* < 0.0001; oxycodone-treated: *F* = −1.15e-05 *p* = 0.0189) (Figures 1A,B).

We repeated the FLD assays at 6 dpf, 24 h after the end of exposure. We found no significant differences between the conditions in basal locomotion or in the light period. During the light period, amphetamine- and nicotine-treated fish showed faster rates of recovery than untreated fish (*F* = −1.20e-06, *p* < 0.0001; *F* = −5.97e-07, *p* = 0.0014, respectively). There was a significant difference in locomotion during the dark period, where amphetamine-treated fish moved more than controls (*p* = 0.0168). There were no significant differences in behavior following the short exposure to light, either in startle response or the following recovery (Figures 1C,D).

Additionally, to determine whether 1–5 dpf drug exposure may have affected the development of pathways involved in the control of behavior, we compared FLD responses in the presence of the drugs following developmental exposure with responses following acute (60 min) drug exposure prior to, and during, the assay. Qualitatively similar results were seen for the majority of measures (see Supplementary Table 3; Supplementary Figure 1). However, acute exposure to oxycodone significantly reduced basal locomotion compared to both control (*p* = 0.0025) and larvae developmentally exposed to oxycodone (*p* = 0.0033). Differences in movement between acute and developmental exposure were persistent in light and dark periods (*p* < 0.0001, *p* = 0.0421, respectively). However, there was no difference in locomotion between either acutely or developmentally oxycodone-treated larvae and controls in light and dark periods. Similarly to oxycodone-treated fish, although both acute and developmental exposure to amphetamine significantly reduced basal locomotion relative to control (*p* < 0.0001 for both), significantly greater reduction in locomotion was seen following acute rather than developmental exposure (*p* = 0.0468). Differences in movement compared to controls were persistent in light and dark periods for acute exposure (*p* < 0.0001, *p* = 0.0053, respectively) but only in the light period for developmental exposure (*p* = 0.0278). No differences in locomotion were observed for nicotine-treated fish at any stage of the assay.

All treated groups showed faster than control rates of recovery during the light period (*p* < 0.0001 for all). There was also an effect of length of exposure, where oxycodone developmentally exposed fish recovered more slowly than acutely exposed larvae (*p* = 0.0513), but the opposite could be seen following nicotine exposure, where developmentally exposed larvae recovered more quickly (*p* = 0.0468).

There was no significant difference in startle response magnitude following 1 min light exposure for any treated fish. However, in the dark period following short light exposure, fish acutely exposed to oxycodone (*p* = 0.0125), and acutely and developmentally exposed to amphetamine, move significantly less than controls (*p* < 0.0001, *p* = 0.0309, respectively). Acute oxycodone treatment results in a decrease in movement compared to developmental oxycodone exposure (*p* = 0.0031). Additionally, when looking at rate of recovery during the dark period, amphetamine-treated groups recover significantly faster than controls (acute: *p* = 0.0246; developmental: *p* < 0.0001), in contrast to fish acutely exposed to nicotine, which recover more slowly than controls (*p* = 0.0008). Developmental exposure to these two drugs leads to significantly faster recovery when compared to acute exposure (amphetamine: *p* = 0.0001; nicotine: *p* < 0.0001). There was no difference in recovery of oxycodone-treated animals (Supplementary Figure 1).

### Drug Exposure Affects Habituation to Acoustic Startle

As response to acoustic startle is also used as a measure of anxiety (67) and habituation to acoustic startle is sensitive to modulation by drugs of abuse, including nicotine, amphetamine and opioids, we assessed the impact of developmental exposure on acoustic startle in larval fish.

**Figure 2:**
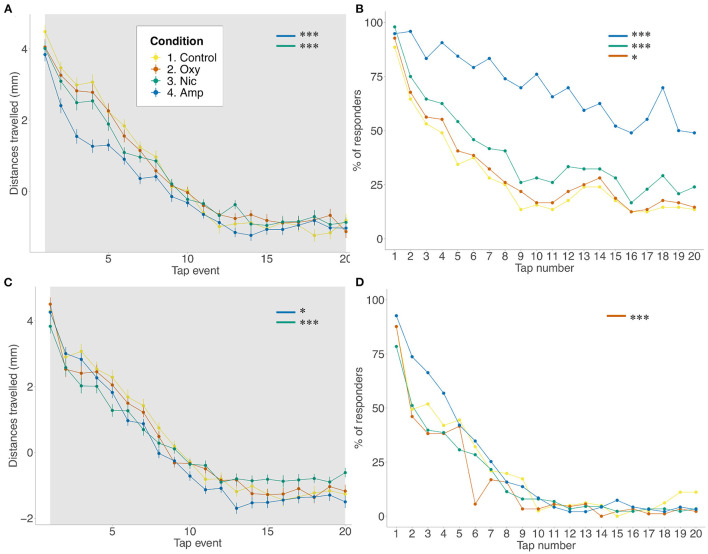
Differences in habituation to acoustic startle. **(A,B)** Acoustic startle assay at 5 dpf in the presence of the drug. **(C,D)** Acoustic startle at 6 dpf in the absence of the drug. Tapping sound is delivered every 2 s. **(A,C)** Habituation to acoustic stimuli by larvae from each group during acoustic stimuli events. Data shows mean ± SEM. Data was fitted to the linear mixed effect model and *post-hoc* Tukey test was used to identify multiple correlations. **(B,D)** Proportion of responders for each stimulus event. Beta regression and likelihood ratio tests were performed to assess the interaction between the tap event number and condition. *Post-hoc* Tukey test was used to identify multiple correlations. Sample size *n* = 48 for each group (*p < 0.05, ***p < 0.001 Tukey's multiple comparison test).

As seen for FLD, there was a reduction in distance traveled in the basal period for amphetamine- and nicotine-treated fish (*p* < 0.001; *p* < 0.001, respectively) compared to controls, but not for oxycodone-treated fish. The same changes were seen across tap stimuli (amphetamine: *p* < 0.001; nicotine: *p* < 0.001) There was a significant effect of treatment on rates of habituation as assessed by distance traveled such that amphetamine and nicotine fish habituated more slowly than controls (*F* = 0.042538, *p* < 0.0001; *F* = 0.018232, *p* < 0.0001, respectively) (Figure 2A). A similar effect of treatment was seen when looking at the proportion of responders analysis. Due to observed differences in locomotion, for the number of responders analysis, habituation to the acoustic stimuli was quantified as the proportion of fish moving more than 2^*^SD above the mean, condition-specific, baseline threshold values. Using the proportion of responders criteria, 88% of control animals responded to the first tap and 13% to the last in line with the habituation response paradigm (40). Treated animals showed significantly reduced habituation such that more treated individuals than control individuals responded to the last tap stimuli (amphetamine- and nicotine-treated fish: *p* < 0.001; oxycodone-treated fish: *p* = 0.0163) (Figure 2B).

At 6 dpf, following 24 h of withdrawal, there was a significant effect of treatment on movement during the baseline period such that amphetamine-, nicotine- and oxycodone-treated fish moved less than controls (amphetamine-treated fish: *p* = 0.0083; nicotine- and oxycodone-treateds fish: *p* < 0.001) (Figure 2C). However, during the tap events only nicotine treatment resulted in decreaseds locomotion (*p* = 0.0081). Following exposure to acoustic stimuli, there was a significant effect of condition on ratess of habituation such that amphetamine and nicotine fish habituated faster than controls (*F* = −0.012488, *p* = 0.0179; *F* = −0.029279, *p* < 0.0001, respectively). There was also a significant effect of condition on proportion of responders for oxycodone-treated fish (*F* = 0.0480, *p* = 0.0002), where exposed larvae habituated faster than unexposed fish (Figure 2D).

### Developmentally Exposed Fish Display Less Exploratory/Bold Behavior

Next, we tested whether drug exposure affected social and exploratory/bold behavior using an established sociability assay (41), as these endophenotypes are associated with psychiatric disorders (1).

There were no differences in time spent with conspecifics between exposed and unexposed fish (Supplementary Figure 2). There was a non-significant trend for a reduction in the number of exploratory/bold individuals following developmental exposure to drugs (*p* = 0.0846). Control fish had the highest percentage of exploratory/bold individuals at 59.72%, with 46.48% for amphetamine, 50% for nicotine and 51.39% for oxycodone.

Taken together our behavioral experiments show a clear effect of drug exposure on behavior which are consistent with mammalian systems. Our results show evidence of possible adaptation to the presence of the drug: we see differences in response following acute vs. chronic drug exposure as well as following acute withdrawal.

### RNA Sequencing Shows Common and Drug-Specific Changes in Gene Expression in Response to Developmental Exposures

**Figure 3:**
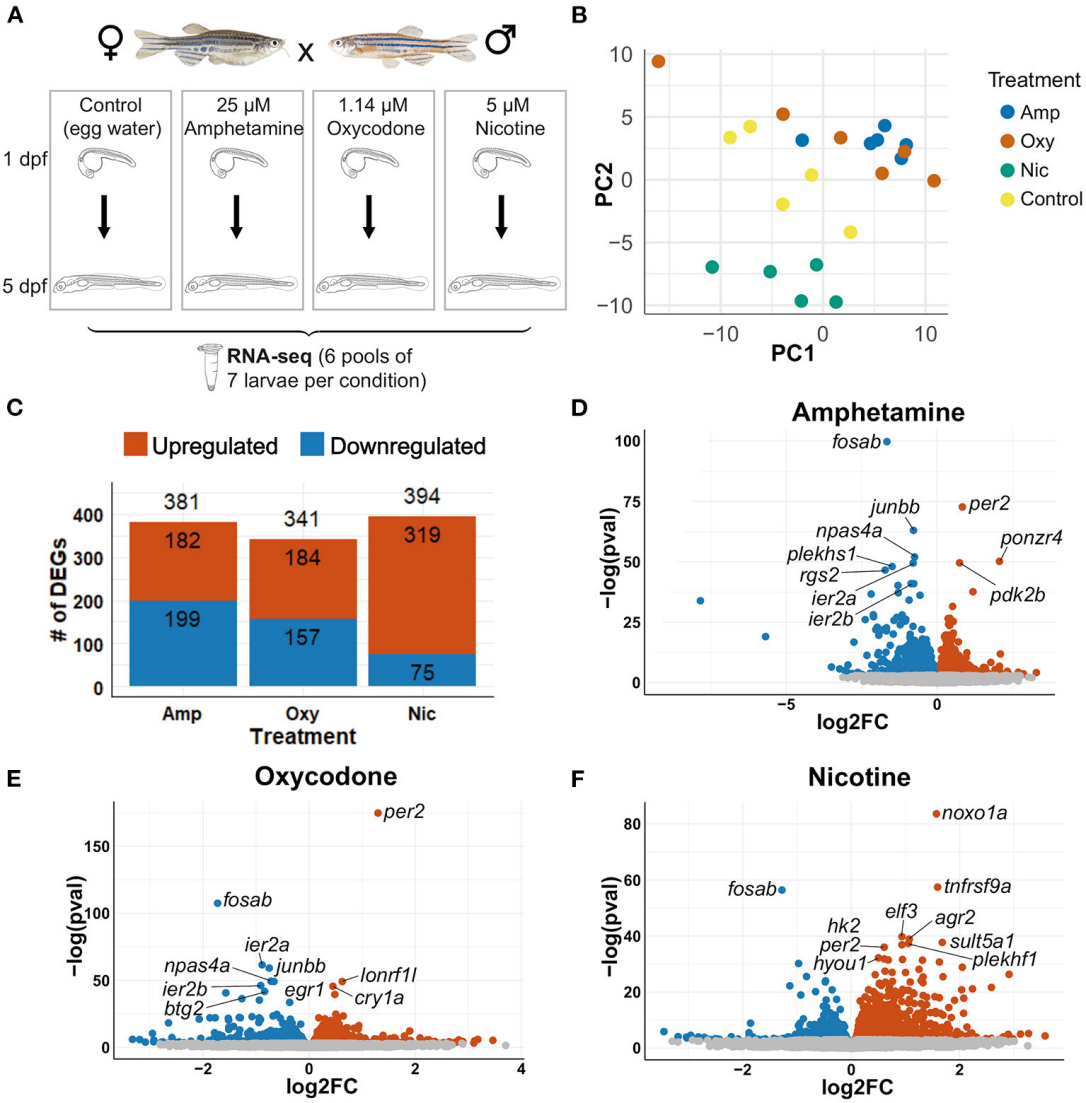
Differential gene expression analysis. **(A)** Schematic of the experimental design. Zebrafish were exposed to nicotine, oxycodone and amphetamine or left untreated from 1 to 5 dpf before being collected in six pools of seven larvae per condition for RNA-seq. **(B)** PCA of samples that passed all quality control checks. Samples from each condition group together. Amphetamine and oxycodone samples are clustered more closely (and so have more similar expression profiles) than nicotine samples. **(C)** Bar chart showing the number of differentially expressed genes (DEGs) in each drug treatment. Upregulated genes are shown in red and downregulated genes are in blue. **(D–F)** Volcano plots showing the distribution of DEGs. Genes that are not significant (adjusted *p*-value ≥ 0.05) are in gray, significant genes (adjusted *p*-value < 0.05) are in red if upregulated and in blue if downregulated. The top 10 DEGs by *p*-value are labeled in each plot.

To investigate the changes in transcriptional profiles and biological pathways of drug-treated larvae, we performed RNA-seq on wild-type zebrafish larvae exposed to 5 μM nicotine, 1.14 μM oxycodone and 25 μM amphetamine from 1 to 5 dpf, along with unexposed controls (Figure 3A). Principal component analysis (PCA) showed, as expected, that the samples that passed all quality control checks cluster according to the treatment (Figure 3B).

Differential gene expression analysis was carried out with an adjusted *p*-value threshold of 0.05. Developmental exposure to amphetamine, oxycodone and nicotine caused differential expression of 381, 341, and 394 genes, respectively (Figure 3C). The distribution of differentially expressed genes (DEGs) for each drug treatment is shown in Figures 3D-F.

**Figure 4:**
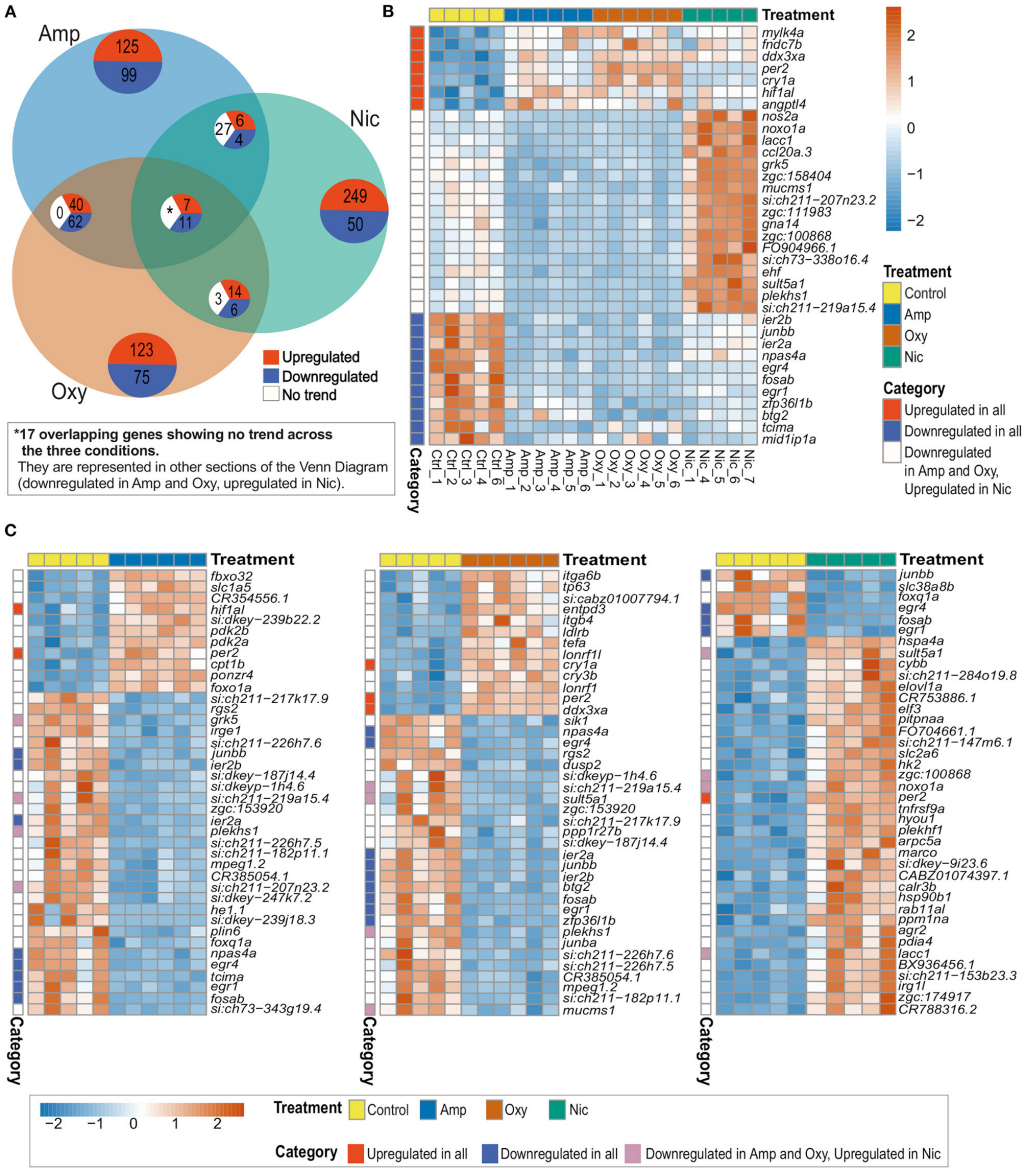
Comparison of DEGs across treatments reveals both common and distinct changes in gene expression. **(A)** Venn diagram showing the number of overlapping DEGs across treatments. Upregulated genes are in red, downregulated genes in blue and genes showing no overlapping trend in comparisons are shown in white. The asterisk (*) represents 17 of the overlapping DEGs with no overlapping trend across the three conditions. These genes are represented in other sections of the Venn Diagram. **(B)** Heatmap showing the expression of 35 overlapping DEGs across all treatments. Overlapping DEGs are shown in three categories: upregulated in all treatments, downregulated in all treatments and downregulated in amphetamine and oxycodone but upregulated in nicotine treatment. **(C)** Heatmaps showing the top 40 DEGs in amphetamine-, oxycodone- and nicotine-treated larvae. Overlapping DEGs are shown in three categories as in **(B)**. Each row/gene of each heatmap has been centered and scaled (mean = 0, standard deviation = 1).

Thirty five genes are differentially expressed across all three treatments; seven of these overlapping DEGs are upregulated and 11 are downregulated across all drug treatments, whereas 17 genes are downregulated in amphetamine and oxycodone but upregulated in nicotine treatment (Figures 4A–C). There is a larger overlap of DEGs between amphetamine and oxycodone treatments than between amphetamine and nicotine treatments which is surprising given both amphetamine and nicotine are stimulants and oxycodone is an opioid. Amphetamine and oxycodone treatments share a total of 120 common DEGs, 47 of which are upregulated and 73 of which are downregulated in both sets of treatments (Figure 4A). Amphetamine and oxycodone samples also cluster together in the PCA (Figure 3B), suggesting their expression profiles are more similar to each other than to nicotine.

Among the common DEGs in response to all developmental drug exposures are genes involved in development, regulation of the circadian rhythm and the immune response. The hypoxia inducible factor, *hif1al* (orthologous to mammalian HIF3A), which is reported to play key roles in developmental morphogenesis (68), is upregulated across all three drug exposures. Two central circadian clock genes, *per2*, period circadian clock 2, and *cry1a*, cryptochrome circadian regulator 1a, are also upregulated across all treatments, suggesting an alteration to the regulation of circadian rhythm.

A set of brain-expressed genes including *fosab* and *junbb* (orthologous to mammalian c-FOS and JUNB), which are implicated in addiction (69, 70), and *npas4a* (orthologous to mammalian NPAS4), which is implicated in reward learning and memory processes in rodents (71), are also downregulated by all three drugs. In addition, early growth response genes *egr1* and *egr4* (EGR1 and EGR4 in mammals), which are involved in numerous biological processes including eye morphogenesis (72), brain development (73) and circadian regulation of gene expression (74), show downregulation across all drug exposures.

Among the group of DEGs which are downregulated in amphetamine and oxycodone but upregulated in nicotine treatment are immune function and metabolism-related genes. For example, *ccl20a.3* (orthologous to mammalian CCL20), which is involved in immune response and leukocyte chemotaxis, is downregulated in amphetamine and oxycodone but upregulated in nicotine treatment. Likewise, *noxo1a*, NADPH oxidase organizer 1a (orthologous to mammalian NOXO1), and *nos2*, nitric oxide synthase 2a (orthologous to mammalian NOS2), show the same trend in differential expression.

### Gene Ontology Term Enrichment: Enrichment for Both Overlapping and Distinct Biological Processes

**Figure 5:**
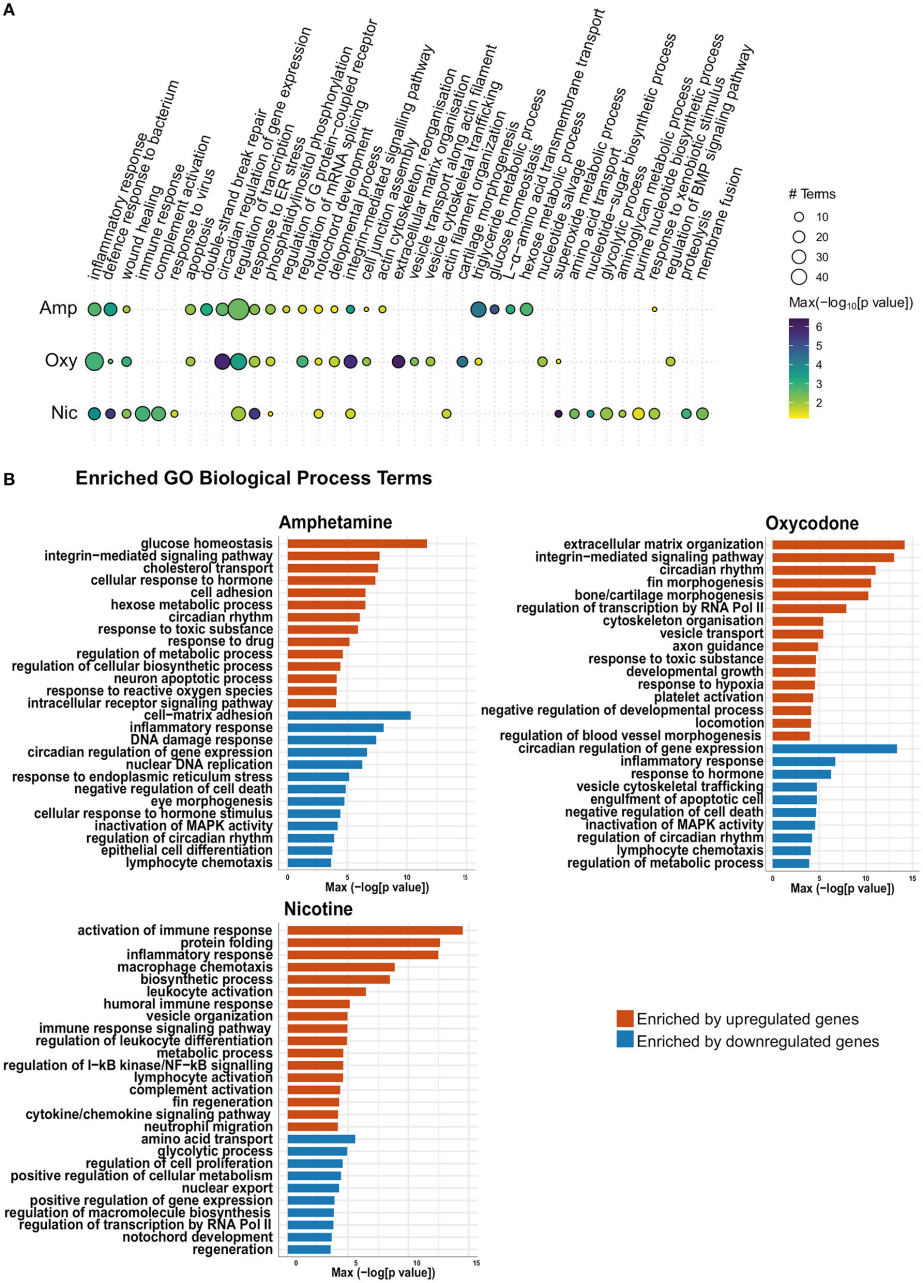
Gene Ontology (GO) term enrichment analysis shows enrichment of both common and distinct biological process terms across treatments. **(A)** Bubble plot of the GO BP enrichment results across the three drug treatments. Individual enriched BP terms were aggregated to a parent term. For example, regulation of circadian rhythm (GO:0042752), circadian rhythm (GO:0007623) and circadian behavior (GO:0048512) are all aggregated to the parent term circadian regulation of gene expression. The size of each circle represents the number of individual terms enriched for each parent term and they are colored by the smallest of the *p*-values (–log10 scale). **(B)** Bar charts showing top 40 upregulated terms and top 40 downregulated terms (by *p*-value) for each drug treatment. Individual enriched BP terms were aggregated to a representative term and colored by the smallest of the *p*-values (–log10 scale) as in **(A)**.

In order to identify the biological pathways that are affected by developmental drug exposures, we analyzed enriched Gene Ontology (GO) biological process terms in differentially expressed gene lists. As expected, this analysis revealed enrichment of both common and drug-specific biological processes, suggesting an overlap of pathways that are affected across treatments as well as drug-specific changes in cellular pathways. A summary of enriched GO biological process terms can be found in Figure 5A, which is split into those enriched by upregulated genes and downregulated genes in Figure 5B.

Across all developmental drug exposures, there is an enrichment for terms related to development of anatomical structures, such as the notochord (GO:0030903), retina (GO:0060041) and the lymphatic vessels (GO:0001945) (Figures 5A,B). These enrichments are driven by differential expression of genes involved in development and morphogenesis, such as *egr1*.

Multiple genes involved in the regulation of circadian rhythm are differentially expressed across all three drug exposures. Accordingly, there is an enrichment of GO terms related to the regulation of circadian rhythm (GO:0007623, GO:0042752, and GO:0032922) in amphetamine and oxycodone treatments. Two central circadian clock genes, *per2* and *cry1a*, are significantly upregulated across all three conditions and contribute to the enrichments of circadian terms in amphetamine and oxycodone treatments.

Enrichment of GO terms related to hypoxia (GO:0036293 and GO:0001666) are prominent in oxycodone and nicotine exposures. The hypoxia-inducible factor, *hif1al* (orthologous to mammalian HIF3A), which is reported to play important roles in developmental morphogenesis (68), is upregulated across all conditions and contributed to the enrichments in oxycodone and nicotine treatments.

Across all drug treatments, genes involved in cell death show differential expression and GO terms related to neuron death and apoptosis are enriched in amphetamine and oxycodone treatments (including GO:0070997, GO:0051402, GO:0043066 in amphetamine and GO:0006915 and GO:0008219 in oxycodone). In amphetamine and oxycodone exposures, genes involved in the regulation of apoptosis (*pycard* and *card14* in amphetamine and *pdcd4b* and *niban2a* in oxycodone) are differentially expressed and drive these enrichments. Even though cell death terms are not enriched, there is a significant change in the expression level of caspases in nicotine treatments; the genes *casp3b, casp8, casp1*, and *casp9* (orthologous to mammalian caspases 3, 8, 1, and 9, respectively) are upregulated in nicotine treatments, suggesting an activation of both intrinsic and extrinsic apoptosis pathways.

Reflecting the larger overlap of differential gene expression between amphetamine and oxycodone exposures, there are some terms which are enriched in amphetamine and oxycodone, but not in nicotine treatments. For example, the term “response to toxic substance” (GO:0009636) is enriched in amphetamine and oxycodone but not in nicotine.

It is noteworthy that some terms that are enriched across all conditions are not driven by the same changes in gene expression. For example, the term “inflammatory response” (GO:0006954) is enriched across all treatments. However, inflammatory genes driving this enrichment are downregulated in amphetamine and oxycodone treatments but upregulated in nicotine treatments (Figure 5B).

Enrichment of some GO terms are drug specific. For example, there is an enrichment for morphogenesis-related terms among upregulated genes in oxycodone treatments (Figure 5B), such as bone morphogenesis (GO:0060349), cartilage morphogenesis (GO:0060536) and chondrocyte differentiation (GO:0002062).

### Developmental Drug Exposures Affect Expression of Innate Immune Genes

Developmental exposures to all three drugs led to differential expression of multiple genes involved in the regulation of the immune system. Accordingly, GO term enrichment analysis shows enrichment of inflammation and immunity-related terms across all treatments. The term “inflammatory response” (GO:0006954) is enriched across all exposures (Figure 5B).

Drugs of abuse, including stimulants such as methamphetamine and cocaine, and opioids such as morphine, are reported to have immunomodulatory effects and increase susceptibility to infectious diseases (75–78). Our transcriptomic analysis showed a downregulation of genes involved in immune response and inflammation in amphetamine and oxycodone treatments. The genes *ccl20b* and *ccl20a.3* (both orthologous to mammalian CCL20) and *nos2a* (orthologous to mammalian NOS2) are significantly downregulated in both sets of treatments, leading to enrichment of the term “inflammatory response” (GO:0006954). The gene *irg1l* (orthologous to mammalian ACOD1) is also among the downregulated genes driving this enrichment in amphetamine. Several other genes, including *tnfsf10* and *cd180*, are also downregulated in amphetamine treatments and collectively lead to enrichment of the term “immune system process” (GO:0002376).

In contrast, pro-inflammatory immune genes such as *il1b, il6st, nos2a*, and *noxo1a* (orthologous to mammalian IL1B, IL6ST, NOS2, and NOXO1) are upregulated in nicotine treatments, suggesting an activation of the immune response. Accordingly, several terms including inflammatory response, activation of the immune response and defense response (GO:0006954, GO:0002253 and GO:0006952, respectively) are enriched by upregulated immune genes in nicotine treatments.

### Developmental Drug Exposures Lead to Region-Specific Differential Expression of Immediate-Early Genes

**Figure 6:**
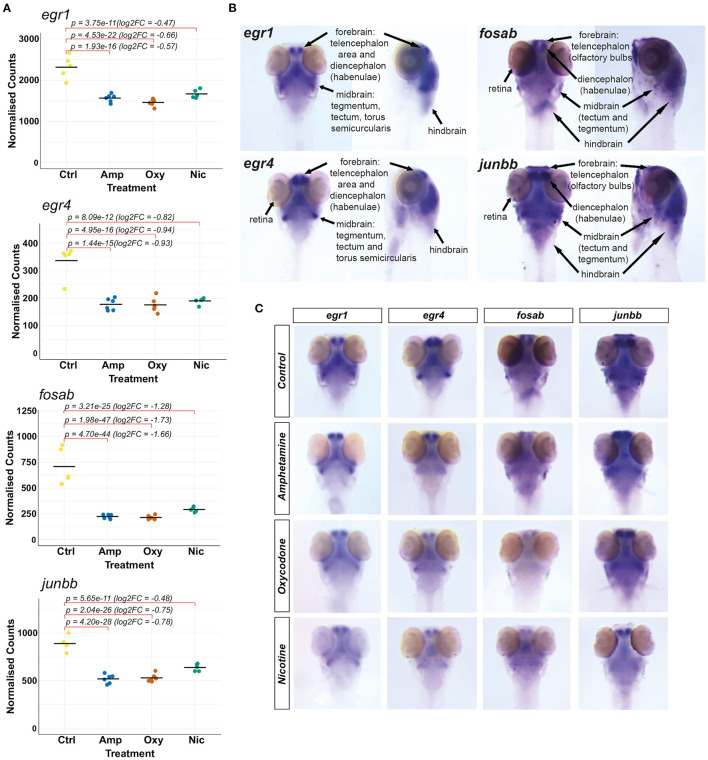
Investigating changes in spatial expression patterns of IEGs which are downregulated in response to developmental exposures. **(A)** Plots of normalized counts for the candidate genes chosen for mRNA *in situ* hybridization: *fosab, junbb, egr1*, and *egr4*. **(B)** Annotation of stained neuroanatomical regions in untreated 5 dpf larvae for *egr1, egr4, fosab*, and *junbb*. The zebrafish larval brain anatomical atlas (79), schematics of the developing zebrafish brain (http://zebrafishucl.org/zebrafishbrain) and whole-mount mRNA ISH staining images of other genes expressed in distinct neuroanatomical regions at 5 dpf were used as reference for the annotation. **(C)** Dorsal images of whole-mount mRNA ISH staining of candidate genes in drug-exposed larvae and untreated controls. From left to right: *egr1, egr4, fosab, junbb*. Lateral images can be found in Supplementary Figure 3.

Our transcriptomic analysis shows that the IEGs *fosab, junbb, egr1* and *egr4* (orthologous to mammalian c-FOS, JUNB, EGR1 and EGR4, respectively) are significantly downregulated in response to developmental drug exposures (Figure 6A). These genes are associated with synaptic plasticity and they are implicated in brain development (73), memory consolidation (80), neurodegenerative diseases, addiction and other psychiatric disorders (81). Therefore, we were interested in investigating drug-induced changes to the spatial expression pattern of *fosab, junbb, egr1*, and *egr4* in zebrafish using whole-mount mRNA ISH staining.

We found that all four genes have overlapping expression patterns and are expressed in the forebrain (both telencephalic and diencephalic areas), in the midbrain (tectum and tegmentum) and the hindbrain of untreated 5 dpf larvae (Figure 6B). The genes *egr1* and *egr4* are also expressed in the torus semicircularis of the midbrain and the retina is stained for *egr4, fosab* and *junbb* transcripts.

Reflecting the downregulation of transcripts seen in RNA-seq (Figure 6A), mRNA hybridization and staining intensity for all four transcripts is reduced across all neuroanatomical regions (Figure 6C; Supplementary Figure 3). Among other regions, we found a reduction in the expression of all four genes in the habenulae and the tegmentum, which are implicated in reward processing, addiction (82) and dementia (83), as well as the optic tectum. In particular, we found that the expression of *fosab* and *junbb* is diminished in the olfactory bulbs, a key area implicated in alcohol preference (84, 85), depression (86) and schizophrenia (87).

### Developmental Drug Exposures Lead to Differential Expression of Genes Involved in the Regulation of the Circadian Rhythm

Substance use has been reported to alter the circadian rhythm (88), which predisposes individuals to a variety of psychiatric conditions including depression (89, 90), bipolar disorder (91) and addiction (92–97). We found that developmental exposures to all three drugs led to differential expression of multiple genes involved in the regulation of circadian rhythm and related GO terms are enriched in amphetamine and oxycodone treatments (including GO:0007623, GO:0042752, and GO:0032922).

**Figure 7:**
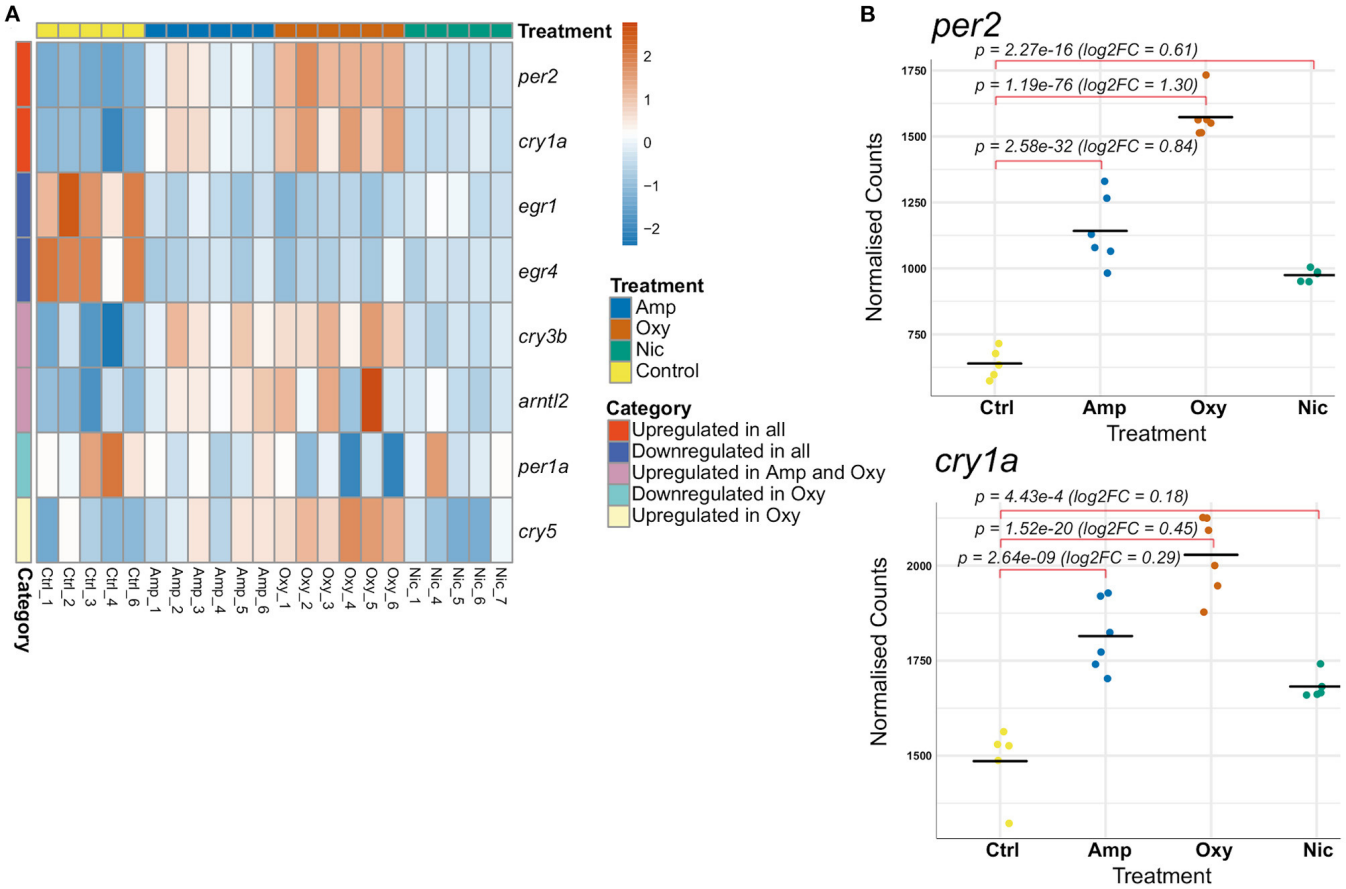
Developmental drug exposures lead to differential expression of circadian regulators. **(A)** Heatmap showing expression levels of significantly differentially expressed genes involved in the regulation of circadian rhythm across treatments. Each row/gene of the heatmap has been centered and scaled (mean = 0, standard deviation = 1). **(B)** Plots of normalized counts for the genes *per2* and *cry1a*, which are significantly upregulated in all treatments.

A total of eight genes involved in the regulation of circadian rhythm were differentially expressed, some of which are common whilst others are drug-specific (Figure 7A). Two central circadian clock regulators, *per2* and *cry1a*, are significantly upregulated (Figure 7B) while *egr1* and *egr4*, which are implicated in regulating some components of the circadian clock (74), are significantly downregulated across all treatments, suggesting an overlapping mechanism of circadian disruption. Significant downregulation of *per1a* and upregulation of *cry5* is specific to oxycodone exposures, while *cry3b* and *arntl2* are upregulated both in amphetamine and oxycodone but not in nicotine exposures.

### Changes in Gene Expression Do Not Correlate With Changes in Circadian Rhythm

As RNA-seq analysis showed significant expression differences in genes involved in the circadian cycle, we decided to perform two behavioral assays to determine if changes in expression correlate with changes in behavior in the larvae. First, we looked for differences in the free running period in the dark. At 5–7 dpf, during 54 h in darkness, all groups of developmentally exposed fish moved significantly less than controls (amphetamine: *p* < 0.001; nicotine: *p* = 0.00201; oxycodone: *p* = 0.00105), however no differences in periodicity were observed between exposed fish and controls (Supplementary Figure 4).

Additionally, we assessed responses to dusk and dawn in an assay with gradually changing light and dark periods. Nicotine- and amphetamine-treated fish moved significantly less in the 2-h light period before dusk (*p* < 0.001; *p* = 0.0022, respectively). Similar differences were observed in the 2-h dark period preceding the dawn, when all treated groups showed decreased locomotion compared to controls (amphetamine: *p* < 0.001; nicotine: *p* = 0.0306; oxycodone: *p* = 0.0054). Next, we looked at locomotion and the slope of response during the 46 min of gradual dusk and dawn. All groups of treated fish moved less than untreated fish (*p* < 0.001 for all) during the dusk period. However, the rate of recovery was significantly slower for only amphetamine-treated fish (*F* = 2.26e-05, *p* < 0.001). During dawn, fish exposed to amphetamine showed significantly reduced locomotion compared to controls (*p* < 0.001) and nicotine-treated fish showed a significantly reduced rate of change of response to increasing light compared to controls (*F* = 8.77e-05, *p* < 0.001) (Supplementary Figure 5). No other comparisons were significant.

### RT-qPCR Shows a Long-Lasting Impact in Gene Expression of Circadian Rhythm Regulators

Alterations in the circadian rhythm are reported to predispose individuals to addiction and other psychiatric conditions (89–97) and are associated with relapse in recovering addicts (98, 99). Therefore, we were interested in investigating the persistence of changes in expression of circadian rhythm genes following cessation of substance exposure.

**Figure 8:**
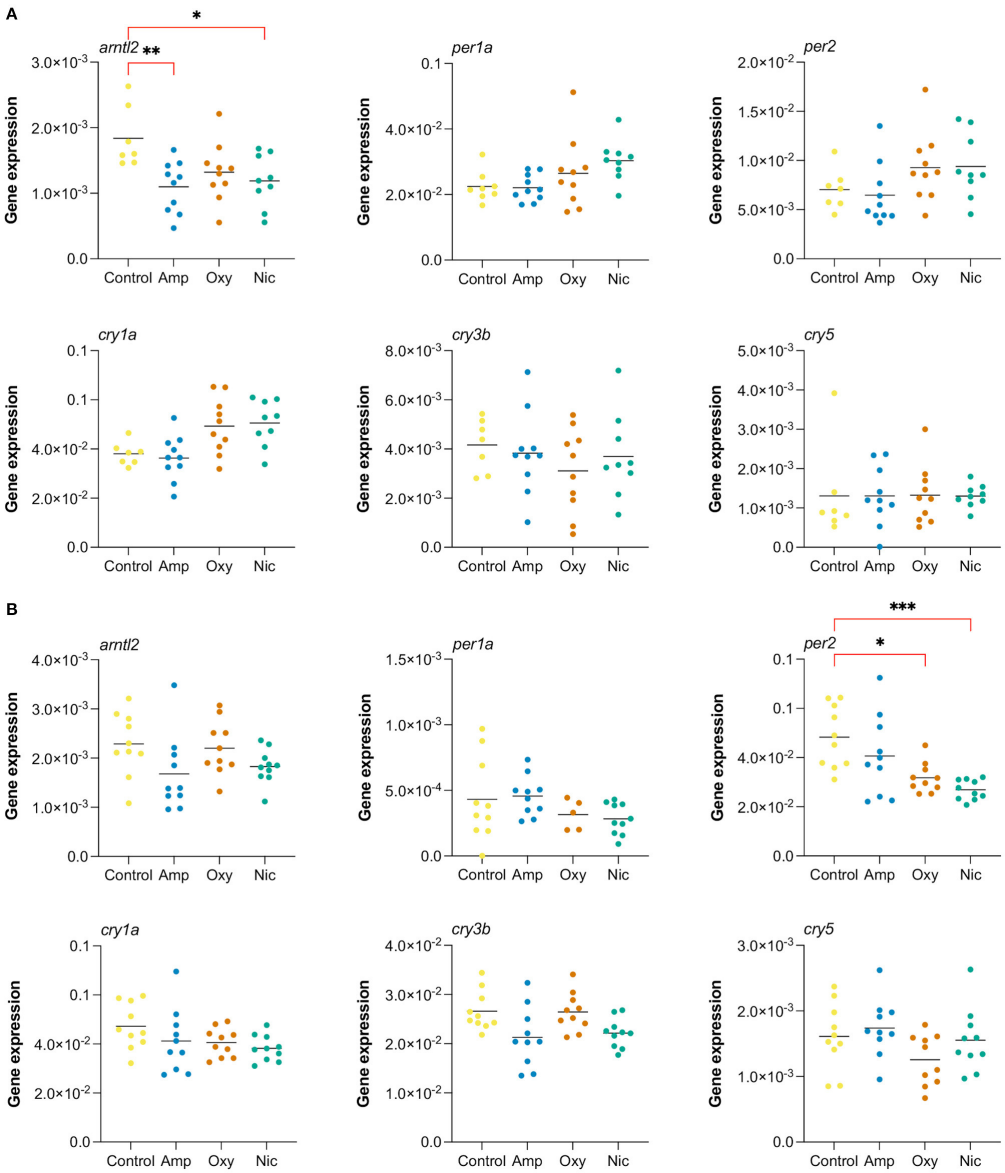
Long-lasting changes in expression of genes involved in circadian cycle. Gene expression analysis of six genes associated with the circadian cycle: *arntl2, per1a, per2, cry1a, cry3b, cry5*. RT-qPCR was performed at **(A)** 7 dpf and **(B)** at 21 dpf (*p < 0.05, **p < 0.01, ***p < 0.001, after Bonferroni multiple test correction).

In order to investigate changes in gene expression at later stages of development, we raised developmentally exposed larvae to 7 and 21 dpf stages in the absence of drugs and collected samples for RT-qPCR. We performed RT-qPCR on six circadian rhythm genes that were found to be differentially expressed at 5 dpf, both common, like *cry1a* and *per2*, and drug-specific, like *arntl2, per1a, cry3b*, and *cry5* (Figure 8).

We found that the gene *arntl2* was differentially expressed at 7 dpf, 2 days after cessation of exposure, in amphetamine- and nicotine-exposed fish. In contrast to the upregulation seen in RNA-seq at day 5, we found a downregulation of *arntl2* at 7 dpf in amphetamine- and nicotine-exposed fish which did not persist at 21 dpf. There were no differences in the expression of *arntl2* in oxycodone-treated fish at either time point. Additionally, we found that the gene *per2* is not differentially expressed at 7 dpf but is downregulated for nicotine- and oxycodone-treated fish at 21 dpf (contrary to the upregulation at 5 dpf), suggesting a long-term impact of developmental drug exposures on the expression of circadian rhythm genes.

## Discussion

We modeled consistent drug usage during pregnancy by exposing wild-type zebrafish larvae to 25 μM amphetamine, 5 μM nicotine or 1.14 μM oxycodone. To be sure drug exposure paradigms were sufficient to cause physiological effects we assessed the impact of drug exposure on larval behavior. We found differences in locomotion, response to FLD transition, and habituation to acoustic startle consistent with the published effects of these drugs in mammalian systems. Following a 5-day exposure, in the presence of the drugs we saw decreases in basal locomotion in FLD and acoustic startle assays for amphetamine- and nicotine-treated fish. In the FLD, all treated groups showed faster recovery in the light, and fish exposed to nicotine or amphetamine, but not to oxycodone, showed smaller startle responses. Additionally, we observed decreased rates of habituation to acoustic startle for all treated groups. These differences were not preserved upon withdrawal of the drug.

Transcriptional profiling of developmentally exposed larvae led to differential expression of over 300 genes for each drug exposure, with 35 shared DEGs across the three drugs, suggesting some commonality in the affected pathways, as well as drug-specific changes in gene expression. Accordingly, Gene Ontology (GO) term enrichment analysis revealed enrichment of common as well as drug-specific biological processes. Among the enriched biological process terms are ones related to development of anatomical structures, innate immunity, regulation of the circadian rhythm and cell death, reflecting the differential expression of multiple genes involved in these processes.

### Drug Exposure Led to Changes in Behavior Consistent With Responses in Mammalian Systems

We assessed the behavior of developmentally exposed fish in the presence of drugs and following a 24-h withdrawal period for evidence of conserved responses to drug exposure. We predicted that drug exposure in larval zebrafish will lead to changes in locomotion and behavior consistent with the effect of the drug in mammalian systems.

In the presence of a drug and following exposure from days 1 to 5 of development, as well as following 1 h of acute exposure, locomotion was decreased in amphetamine-treated fish. Although these results are in contrast with the increased locomotor effect of low dose amphetamine in adult rodents following chronic exposure and in the presence of the drug (100), amphetamine has a dose-dependent effect on locomotion such that low doses cause increased locomotion, and high doses a shift from increased locomotion to stereotypic behavior (101), suggesting that our selected concentration was in the high range for larval zebrafish. Similarly, 1–5 day exposure to nicotine led to a reduction in locomotion that may be explained by desensitization of nicotinic receptors (102) or, possibly, by disruption of motor neuron development; exposure to 15 μM nicotine from 1 to 5 dpf was found to delay development of secondary motor neurons in zebrafish previously (103).

Although zebrafish larvae do not show the full complexity of behavior of adult animals, the FLD transition assay has been frequently used to assess anxiety-like behavior in both zebrafish and rodents (104, 105). We assessed effects of the presence of the drugs on FLD using 3 different measures: by looking at the slope of recovery during a 10-min light period; magnitude of the startle response following a short light exposure; and by rate of change in locomotion (slope) in the dark following a short light exposure. We found that nicotine-exposed animals recover more slowly over the 10-min light period, amphetamine- and nicotine-treated animals show a smaller startle response on light/dark transition following a short light exposure, and all exposed groups showed a decreased rate of recovery following a short 1-min light exposure. Although the magnitude of the “startle response” on light to dark transition is commonly used as a measure of anxiety-like behavior (38) where a reduction in distance traveled is taken as indicative of a reduced anxiety-like response, differences in baseline locomotion preclude this interpretation here. A reduced rate of change in locomotion following forced light transition is consistent with reduced rates of recovery that have been interpreted as increased anxiety-like behavior (46), and would be consistent with mammalian studies (106, 107), but again, differences in basal locomotion makes it difficult to draw conclusions here.

On withdrawal of each drug, no differences in basal locomotion were observed. For analysis of anxiety-related behavior, despite what looks like clear qualitative differences, no consistent differences in behavior were observed. However, we observed a lot of variability in all treatment groups which may reflect increased freezing and darting, an indicator of increased anxiety-like behavior in zebrafish (108, 109). Increased intra-condition variability could also explain the lack of significant differences in startle response. Adult studies have shown altered anxiety-like behavior upon exposure and withdrawal of drugs of abuse including ethanol and nicotine in fish as in mammals (110, 111). Developmental exposure and withdrawal from amphetamine, nicotine or opioids result in increased anxiety at later life stages in human and rodent studies (112–117). However, these studies looked at behavior at adult stages following exposure to drugs throughout the entire period of *in utero* development. As we did not examine behavior at adult stages, we are unable to directly relate our findings to previous data. Further, it is not clear how a 1–5 dpf exposure period relates to human gestation. Thus, differences in the exposure period may contribute to observed differences in response.

Although behavioral responses after 24 h of withdrawal following developmental exposures were not as marked as seen following exposure in adult zebrafish, behavioral responses in the presence of each drug following a 5-day exposure were significantly different from the response in the presence of acute drug exposure. Responses were qualitatively similar in that all acutely and developmentally exposed groups showed a faster rate of recovery in the light and no difference in startle response, and both amphetamine-treated groups showed faster recovery in the dark. However, acute exposure led to a significantly greater reduction for nicotine-treated fish in recovery to light and for amphetamine- and nicotine-treated fish in recovery to dark. A significantly greater rate of recovery to light was observed for oxycodone acutely treated fish compared to developmentally exposed fish. Chronic exposure to a neurotransmitter is frequently associated with reduced sensitivity to subsequent exposures (118) as a result of desensitization, proteolytic cleavage or receptor downregulation. Thus, the observed differences in the effect of acute (1 h) vs. chronic (1–5 dpf) exposure to these drugs of abuse provide evidence of adaptation to the presence of drugs.

We used habituation to acoustic startle to assess sensorimotor gating, a behavior predictive of vulnerability to psychiatric disorders such as schizophrenia and addiction and sensitive to modulation by dopaminergic signaling, in drug-exposed and control larvae (40, 119). Consistent with effects on dopaminergic signaling and acoustic startle in mammalian systems (120, 121), all treated groups habituated more slowly in response to acoustic startle stimuli in the presence of the drug. On withdrawal, larvae that had been exposed to nicotine or oxycodone had increased rates of habituation. This is in contrast to previous rodent studies which reported impaired sensorimotor gating in juveniles following *in utero* exposure to nicotine (122, 123), although different periods of exposure and time points of assessment must be noted. Increased rates of habituation are seen in dopamine receptor D2/D3 loss of function mice (124) and following D2/D3 receptor blockade in both humans and zebrafish (40, 125). Therefore, the increased rates of habituation on drug withdrawal seen here are consistent with reduced D2/D3 signaling, suggesting an effect of drug exposure on development of these pathways.

Thus, we see clear behavioral effects in the presence of the drugs consistent with the known effects of these drugs in mammalian systems. Further, we see significant differences between acute and chronic exposure and on acute withdrawal, suggesting adaptation to the presence of the drugs. It is possible that altered exposure paradigms would impact behavioral outcomes but it is difficult to predict the direction of the effect. For example, other studies of centrally active drugs suggest an intermittent exposure regime increases adaptation (126). However, in line with previous studies in zebrafish (127–129) the behavioral studies provide evidence of conserved physiological effects of drug exposure, supporting the use of this model to examine adaptive effects on gene expression.

To explore the basis of this potential adaptation and subsequent possible long-lasting changes in behavior, we conducted differential gene expression analysis at 5 dpf and examined social behavior at 21 dpf. Altered sociability is associated with a range of psychiatric disorders and has been shown to be correlated with *in utero* drug exposure (1, 130). Here, we did not observe any differences in social behavior in treated vs. untreated animals at the 3-week time point, in contrast to previous evidence from rat studies (131). However, time spent near or away from the stimuli fish in a sociability assay is not only a measure of sociability and can also be considered as an exhibition of exploratory/bold behavior. More adventurous and less scared individuals may be more keen to explore the whole arena of the tank and more willing to leave the “safety” of conspecifics. Although fewer developmentally exposed fish explored the arena away from conspecifics, possibly suggesting increased anxiety, this did not reach significance.

### Developmental Drug Exposures Lead to Differential Expression of Innate Immune Genes in the Larvae, Which Might Have Behavioral Consequences

Drug abuse is reported to have immunomodulatory effects and increase susceptibility to infectious diseases in adults via a range of mechanisms including modification of protective defenses and proinflammatory responses (75–78, 132–134). Even though the effects of drug exposure during development are different from those in adults, as both the CNS and the immune system are still developing, studies have shown increased hospitalization due to infections in the first year of life for amphetamine-exposed children (8). Our transcriptomic analysis shows that developmental exposure to amphetamine and oxycodone downregulates the expression of inflammatory genes such as *ccl20b, ccl20a.3*, and *nos2a*, which may manifest as increased susceptibility to infections in early life. In contrast, several pro-inflammatory genes are upregulated in nicotine treatments, contrary to the anti-inflammatory effect of nicotine seen in adults (135), which might reflect an activation of the neuroimmune system in the brain, associated with addiction and other psychiatric disorders (136, 137).

Neuroinflammation, triggered by drug/alcohol abuse, stress or infections, is characterized by the induction of inflammatory NF-κB and subsequent upregulation of proinflammatory genes, such as IL1B, IL6, TNFA, iNOS, and NOX (136, 137). Studies in humans and mouse models have shown direct links between induction of innate immune genes in the brain and increased susceptibility to attention deficits, addiction and other psychiatric disorders (138–140). Human genetic association studies and post-mortem studies of addicted brains further strengthen the link between addiction and neuroinflammation (141–143). Our transcriptomic analysis shows that pro-inflammatory genes commonly associated with neuroinflammation (*nfkbiz, il1b, il6st, nos2a*, and *noxo1a*) are upregulated in nicotine exposure, which might play a role in attention deficits (4, 8–11) and increased susceptibility to addiction (4) in substance-exposed children.

It is noteworthy that the innate immune system is influenced by the circadian clock and that, in zebrafish, lack of *per2*, which is upregulated in our RNA-seq data, is shown to dampen the innate immune response (144). Upregulation of *per2* might influence the expression of innate immune genes and explain the activation of the inflammatory response seen in nicotine exposure. However, the opposite effect is seen for amphetamine and oxycodone, which also show upregulation of *per2*.

Overall, differential expression of immune genes in response to developmental exposures might influence susceptibility to infectious diseases and also contribute to attention deficits and behavioral dysfunction due to neuroinflammation. However, the impact needs further mechanistic investigation, for example, by using transgenic zebrafish lines to study localization of and quantify immune cells and behavioral assays to investigate prevalence of attention deficits.

### Developmental Drug Exposures Lead to Region-Specific Differential Expression of Immediate Early Genes

The IEGs *fosab, junbb, egr1*, and *egr4*, which are regulators of synaptic plasticity, are significantly downregulated in response to developmental drug exposures. These genes are implicated in addiction, psychiatric disorders and neurodegenerative diseases (81).

Fos and Jun family proteins (including *fosab* and *junbb*) are well-studied in the context of learning, memory and addiction (69, 70, 145). Egr1 has also been shown to be critical for memory consolidation and is downregulated in stress-mediated disorders (146). In fact, reduced mRNA levels of Egr1 are used as a measure of depressive phenotypes (146, 147). Its homolog Egr4 has not been studied in the context of reward processing, but it shows differential expression in addiction, depressive disorders and neurodegenerative diseases (81).

Whole-mount mRNA ISH staining showed that, at 5 dpf, all four genes have overlapping expression patterns and are expressed in the forebrain, midbrain and the hindbrain. *egr1* and *egr4* are also expressed in the torus semicircularis of the midbrain and *egr4, fosab* and *junbb* in the retina. We found a reduction in expression of these genes across all neuroanatomical regions, including brain regions associated with addiction and other psychiatric disorders, which might impact synaptogenesis in these brain regions during development.

All four genes are downregulated in the habenular nuclei of the diencephalon, a region that regulates dopamine levels by GABAergic inhibition of dopaminergic cells in the VTA. ISH staining shows a possible reduction in the size of habenulae, as seen in dementia patients (83) who display abnormal reward behaviors. However, better quantitative methods are needed to estimate habenular size.

The tegmental area of the midbrain, important for relaying inhibitory signals to the thalamus and basal nuclei to prevent unwanted body movements, also shows a downregulation of these genes in response to developmental exposures. Reduced activity in this region might interfere with these inhibitory signals and manifest as symptoms of withdrawal, including tremors and poorly controlled movements seen in substance-exposed newborns, particularly with opioids (148).

Similarly, the optic tectum of the midbrain (superior colliculus in mammals) shows a downregulation of all four genes and the torus semicircularis in the midbrain shows a downregulation of *egr1* and *egr4*. These regions are both important for receiving and processing sensory information and the tectum is also critical for regulating motor outputs such as control and orientation of gaze movements important for attention. Slower rates of information processing and lack of habituation to auditory stimuli is reported in cocaine-exposed newborns (149) and developmentally cocaine-exposed zebrafish have shown altered habituation to visual stimuli and attention (150). Thus, downregulation of IEGs in these regions might underlie reduced synaptogenesis and lead to deficits in information processing and attention reported in substance-exposed children (7, 11).

Strikingly, we found that the expression of *junbb* and *fosab* is diminished in the olfactory bulbs of the telencephalon in drug-exposed larvae. The olfactory bulbs are implicated in psychiatric diseases, for example, their volume is reduced in schizophrenia patients (87). Rats with olfactory bulbectomy are used as a model for depression studies (86) and animal studies have shown a clear impact of alcohol and drug exposures on neuronal circuitries in the olfactory bulbs (84, 85). Further studies are needed to investigate the effect of developmental exposure on the olfactory bulbs, but this has potential implications for reward processing and depression states.

Early intervention to restore the expression of these IEGs could potentially reduce the impact of prenatal drug exposures and the manifestation of associated phenotypes. However, further work is needed for mechanistic characterization of gene function. Zebrafish knockout models and a combination of phenotyping methods, including brain imaging, behavioral assays and RNA sequencing, can be exploited for mechanistic studies.

### Developmental Drug Exposures Induce Differential Expression of Circadian Genes, Some of Which Show Differential Expression at Later Stages

Developmental exposure to amphetamine, oxycodone and nicotine led to differential expression of genes involved in the regulation of circadian rhythm. Alterations in circadian rhythms are implicated in many psychiatric conditions including depression (89, 90), bipolar disorder (91) and addiction (92–97). Therefore, we investigated the behavioral consequences in the larvae and the persistence of expression changes after cessation of drug exposure.

Circadian rhythms are ~24-h, autoregulatory, daily rhythms that regulate the expression of numerous genes involved in a wide range of biological processes, including the dopaminergic and the immune systems. Monoamine oxidase A, which is required for breakdown of dopamine, is the transcriptional target of circadian clock components CLOCK/BMAL1 and PER2 (151), and CLOCK is also shown to inhibit the transcription of tyrosine hydroxylase, the enzyme required for dopamine synthesis (152). In zebrafish, *per2*–/– has also been shown to dampen the innate immune response to LPS (144).

Circadian rhythms can be altered by many factors, including environmental stimuli, genetics and molecular intervention. Given the role of circadian genes in regulating the reward circuitry, these alterations predispose individuals to a variety of psychiatric conditions. Sleep impairments are associated with increased nicotine (95), alcohol and drug consumption (96, 97) and mood disorders (90). A direct impact of circadian genes in periodicity is observed in *per2* knockout zebrafish, which show reduced locomotion under induced light-dark conditions, a 2-h phase delay and lengthened periodicity (153). Similarly, molecular intervention to the clock by drug abuse also alters the circadian rhythm and leads to increased susceptibility to addiction. Daily dopamine agonist injections in mice were reported to entrain the circadian rhythm, which persisted during withdrawal (154). Thus, alterations in sleep cycles following drug abuse, which are associated with relapse following cessation of drug abuse, might be due to persistent changes in the circadian rhythm (98, 99).

We studied the impact of differentially expressed circadian genes on periodicity and phase-shifts (equivalent to sleep/wakefulness cycles in mammals) in developmentally drug-exposed larvae to assess potential shifts in circadian cycles. However, we found that expression changes in circadian rhythm genes do not correlate with changes in periodicity and phase-shifts. As period genes have been linked to altered responses to dusk/dawn in knockout mice (155), we also assessed responsiveness to gradually changing light conditions in exposed larvae. Even though we found a decrease in the rate of response to dusk for amphetamine-treated fish and to dawn for nicotine-treated fish, these results were strongly confounded by significant differences in locomotion and cannot be used to draw conclusions.

Although we saw no clear correlation with behavioral responses in the larvae, besides potentially influencing many circadian-regulated pathways during development, persistent changes in gene expression after cessation of exposure might have long-term effects and predispose individuals to addiction. We investigated such changes in circadian rhythm genes in developmentally exposed fish at 7 and 21 dpf and found long lasting changes in circadian gene expression, which were contrary to the expression changes seen at 5 dpf.

Overall, developmental exposures induce differential expression of circadian rhythm genes, some of which are still seen at later stages of development. Even though this does not correlate with behavioral changes in the larvae, differential expression of these genes might interfere with other circadian-regulated biological pathways during development, such as functioning of the reward and immune systems. It is important to note that differential expression of circadian rhythm genes is reported in other zebrafish RNA-seq experiments (156) and so could be a stress response rather than a drug-evoked response. Glucocorticoid release following stress is also found to induce Per2 expression and cause circadian phase delay (157).

## Conclusions

To conclude, exposure of larval zebrafish to amphetamine, oxycodone and nicotine led to changes in behavior consistent with mammalian systems. Exposure during the period of major organ system development affected locomotion and habituation. However, these differences were most prevalent in the presence of the drug. Differences in acute and developmental effects of the drug are an indicator of adaptation to the presence of the drug.

Whole organism RNA-seq on drug-exposed larvae revealed differential expression of numerous genes and alterations in many pathways, including those related to innate immunity, immunosuppression, neuroinflammation and circadian rhythms, the latter of which were shown to persist after developmental exposure but did not correlate with behavioral changes. Immediate early genes associated with synaptic plasticity were downregulated across all treatments and this was confirmed to occur, among others, in brain regions implicated in reward processing and addiction by *in situ* hybridization. Differential expression of highly localized transcripts is not always picked up by whole organism RNA-seq if the change is constrained to a small group of cells. This might explain the absence of components of neurotransmitter pathways, which have been shown to be differentially expressed in other studies and might explain the possible adaptation to the presence of drugs we observed. However, we anticipate that these early changes in gene expression in the larvae in response to drug exposures are likely to contribute to the consequences of prenatal exposure and their discovery might pave the way to therapeutic intervention to ameliorate the long-lasting deficits.

## Data Availability Statement

The datasets presented in this study can be found in online repositories. The names of the repository/repositories and accession number(s) can be found in the article/Supplementary Material.

## Ethics Statement

The animal study was reviewed and approved by the Animal Welfare and Ethical Review Body at the University of Cambridge (project license P597E5E82) and by Animal Care and Use Ethics Committee at Queen Mary University of London (project license P6D11FBCD and PP9581087).

## Author Contributions

AM and MM conducted experiments, analyzed and interpreted data, and wrote the manuscript. WH conducted experiments. M-TT conducted experiments and analyzed data. IS and RW analyzed and interpreted data. EB-N secured funding and directed and interpreted the gene expression analysis. CB conceived and oversaw the study, interpreted data, and secured funding. All authors contributed to the article and approved the submitted version.

## Funding

This work was funded by the National Institute of Health (NIH U01 DA044400-03, AM, MM, IS, WH, EB-N, and CB).

## Conflict of Interest

The authors declare that the research was conducted in the absence of any commercial or financial relationships that could be construed as a potential conflict of interest.

## Publisher's Note

All claims expressed in this article are solely those of the authors and do not necessarily represent those of their affiliated organizations, or those of the publisher, the editors and the reviewers. Any product that may be evaluated in this article, or claim that may be made by its manufacturer, is not guaranteed or endorsed by the publisher.
