# Supplementary material for: Behavioral and Gene Regulatory Responses to Developmental Drug Exposures in Zebrafish
Source: Front Psychiatry. 2022 Jan 10;12:795175. doi: 10.3389/fpsyt.2021.795175 (PMC8785235; doi:10.3389/fpsyt.2021.795175)
Supplement: Supplementary file 1 [file Data_Sheet_1.docx]

1. **Supplementary Tables**

**Supplementary Table 1.** **Oligonucleotide sequences used for in-situ hybridisation.** T7 promoter sequence in brackets.

| **Primer** | **Sequence** |
| --- | --- |
| fosab-201_left | 5’ CAGCTCCACCACAGTGAAGA |
| fosab-201_right | 5’ (TAATACGACTCACTATAGGG)GCAAACAATTCGCAAGTTCA |
| junbb-201_left | 5’ TCTGTTGGGTTACGGTCACA |
| junbb-201_right | 5’ (TAATACGACTCACTATAGGG)CGAGTGTCGTTCAGTTGTCG |
| egr1-201_left | 5’ CTTCGAATCCCATCCTTTCA |
| egr1-201_right | 5’ (TAATACGACTCACTATAGGG)AGCATTCCGACTCCTCAAGA |
| egr4-201_left | 5’ CATGCAGCAGAAGACTGAGC |
| egr4-201_right | 5’ (TAATACGACTCACTATAGGG)CACAAGGCATTTCATTGCAC |

**Supplementary Table 2.** **Oligonucleotide sequences used for RT-qPCR.**

| **Primer** | **Sequence** |
| --- | --- |
| arntl2 _left | 5’ GGAGCTGCCCAGGAAGAG |
| arntl2_right | 5’ GCATCGTCCTCCATATCCTCA |
| per1a_left | 5’ CGTTACGTCAGAGTACACCCT |
| per1a_right | 5’ AGACAGAAATGACACCGCCA |
| per2_left | 5’ GGAGATCGACAGCATAACCTCT |
| per2_right | 5’ GATGAGAGACACGGCCACTG |
| cry1a_left | 5’ TATTGCCCTGTGAGCTTCGG |
| cry1a_right | 5’ AAAACCCCTTAAGACTGGCAGA |
| cry3b_left | 5’ CCCCAACGGAGACTTCATCA |
| cry3b_right | 5’ GGCGTTCCAGGGGTCATAAA |
| cry5_left | 5’ CTTCTGGTGGAGGCTCGC |
| cry5_right | 5’ GCCATGCAGAGAAACCGGA |

**Supplementary Table 3.** **Multiple comparison of acutely versus developmentally exposed fish in FLD assay.** Significant differences are marked with green. A = acute exposure, D = developmental exposure.

|  |  |  | Control | Amp D | Nic D | Oxy D |
| --- | --- | --- | --- | --- | --- | --- |
| Baseline | Movement | Amp A | < 0.001 | 0.0468 |  |  |
|  |  | Amp D | < 0.001 |  |  |  |
|  |  | Nic A | 0.4172 |  | 0.9998 |  |
|  |  | Nic D | 0.6667 |  |  |  |
|  |  | Oxy A | 0.0026 |  |  | 0.00312 |
|  |  | Oxy D | 1.0000 |  |  |  |
| Light | Movement | Amp A | < 0.001 | 0.6574 |  |  |
|  |  | Amp D | 0.0278 |  |  |  |
|  |  | Nic A | 0.2911 |  | 0.8636 |  |
|  |  | Nic D | 0.9674 |  |  |  |
|  |  | Oxy A | 0.1731 |  |  | < 0.001 |
|  |  | Oxy D | 0.4635 |  |  |  |
|  | Slope of recovery | Amp A | <0.001 | 0.4177 |  |  |
|  |  | Amp D | <0.001 |  |  |  |
|  |  | Nic A | <0.001 |  | 0.0468 |  |
|  |  | Nic D | <0.001 |  |  |  |
|  |  | Oxy A | <0.001 |  |  | 0.0513 |
|  |  | Oxy D | <0.001 |  |  |  |
| Dark | Movement | Amp A | 0.0053 | 0.5440 |  |  |
|  |  | Amp D | 0.5356 |  |  |  |
|  |  | Nic A | 0.9999 |  | 0.9999 |  |
|  |  | Nic D | 1.0000 |  |  |  |
|  |  | Oxy A | 0.7372 |  |  | 0.0421 |
|  |  | Oxy D | 0.7473 |  |  |  |
|  | Slope of recovery | Amp A | <0.001 | 0.6135 |  |  |
|  |  | Amp D | <0.001 |  |  |  |
|  |  | Nic A | <0.001 |  | <0.001 |  |
|  |  | Nic D | 0.2475 |  |  |  |
|  |  | Oxy A | 0.8454 |  |  | <0.001 |
|  |  | Oxy D | <0.001 |  |  |  |
| Jump | Movement | Amp A | 0.7947 | 0.9037 |  |  |
|  |  | Amp D | 1.0000 |  |  |  |
|  |  | Nic A | 0.9958 |  | 0.7429 |  |
|  |  | Nic D | 0.9765 |  |  |  |
|  |  | Oxy A | 0.9999 |  |  | 0.9893 |
|  |  | Oxy D | 0.9995 |  |  |  |
| Dark 2 | Movement | Amp A | <0.001 | 0.1850 |  |  |
|  |  | Amp D | 0.0309 |  |  |  |
|  |  | Nic A | 0.9971 |  | 0.9999 |  |
|  |  | Nic D | 0.9771 |  |  |  |
|  |  | Oxy A | 0.0125 |  |  | 0.0031 |
|  |  | Oxy D | 0.9998 |  |  |  |
|  | Slope of recovery | Amp A | 0.0246 | 0.0001 |  |  |
|  |  | Amp D | <0.001 |  |  |  |
|  |  | Nic A | 0.0008 |  | <0.001 |  |
|  |  | Nic D | 0.2726 |  |  |  |
|  |  | Oxy A | 0.5961 |  |  | 0.8206 |
|  |  | Oxy D | 0.9998 |  |  |  |

1. **Supplementary Figures**


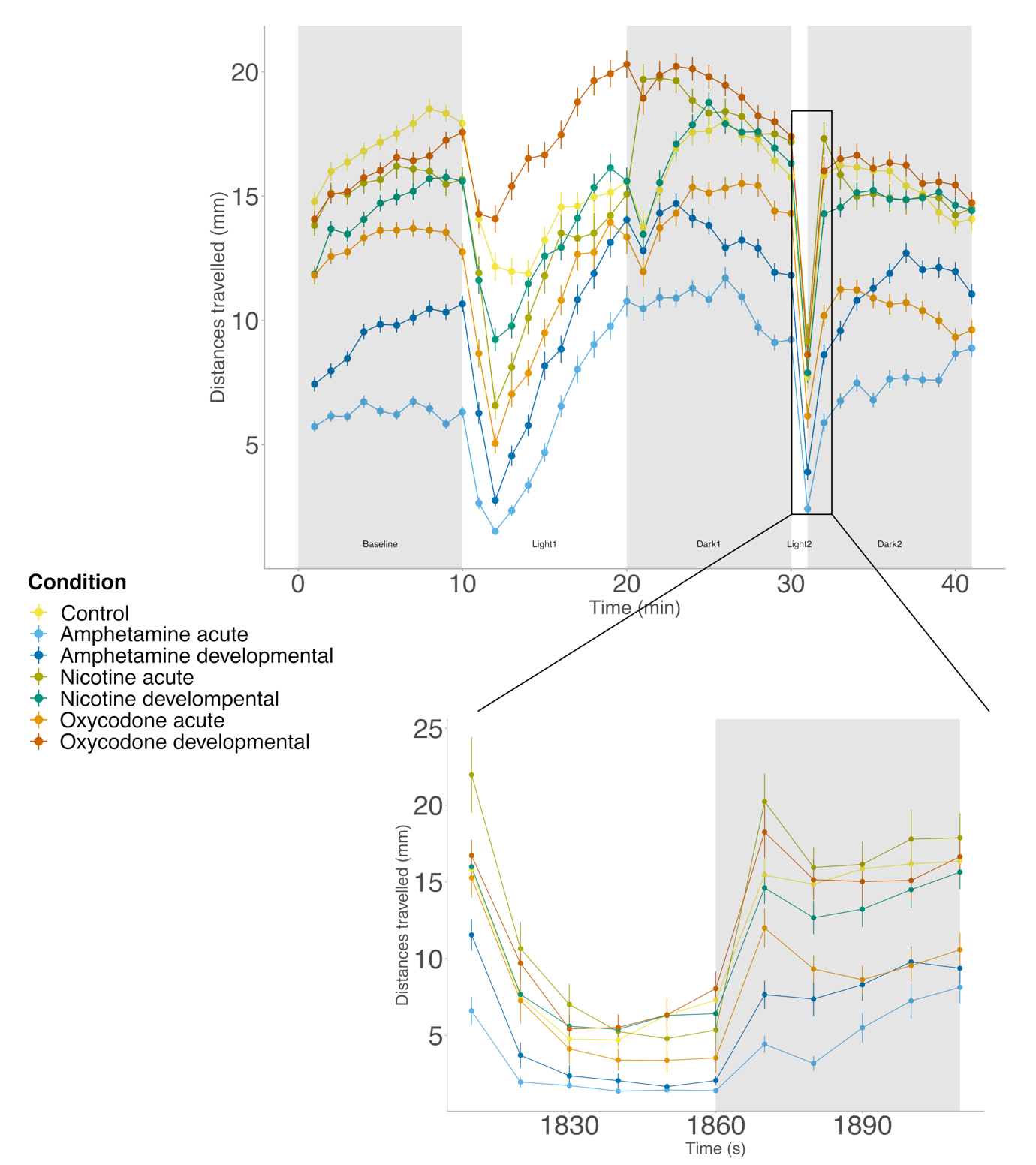


**Supplementary Figure 1. Differences in FLD following acute and developmental exposure.** Mean distance travelled per minute by larvae from each treatment group during alternating dark (grey) and light (white) periods. Startle response following 1 min light exposure is displayed in a 10-second resolution plot. Sample size n=24 for each group. Data shows mean ± SEM.

**
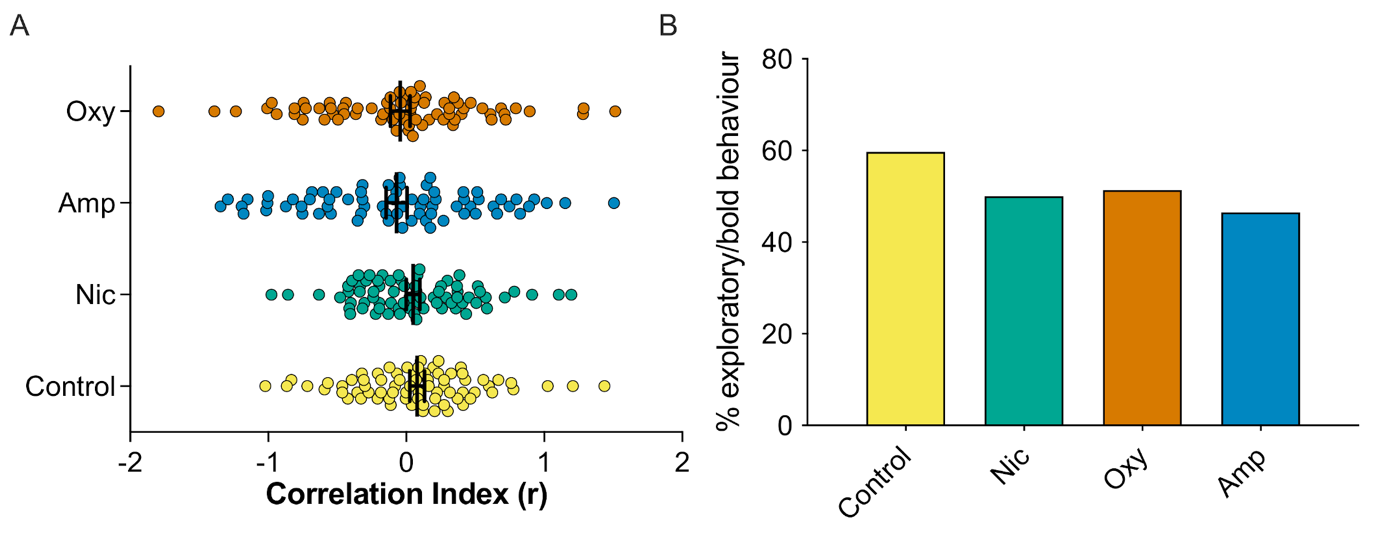
**

**Supplementary Figure 2. Sociability and exploratory/bold behaviour at 3 weeks’ time-point. A)** Sociability correlation index by condition at 3-week time point (20-22dpf). **B)** Percentage of individuals displaying exploratory/bold behaviour at 3-week time point. Sample size n=72 per each group.


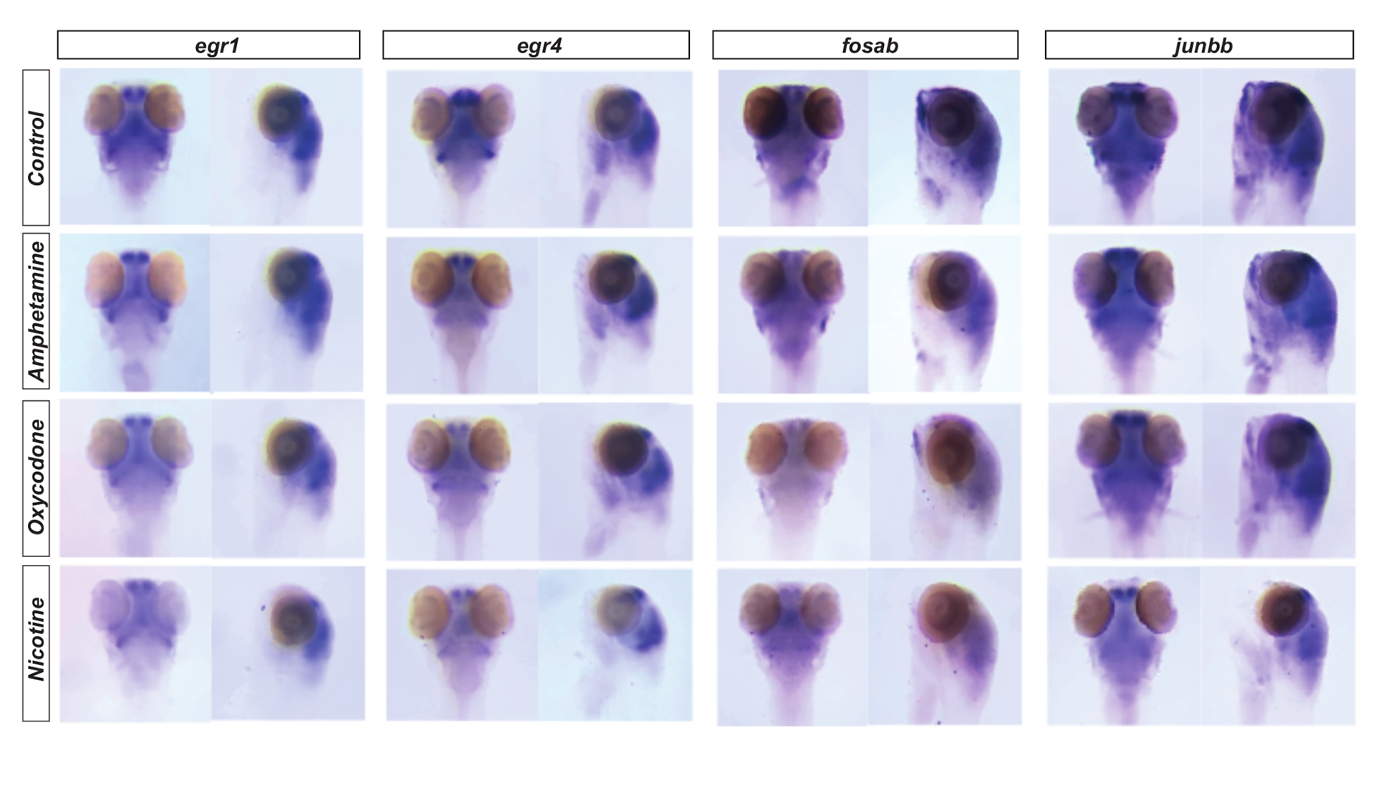


**Supplementary Figure 3.** **Dorsal and lateral images of whole-mount mRNA ISH staining of candidate genes in drug-exposed larvae and untreated controls.** From left to right: *egr1*, *egr4*, *fosab*, *junbb*.


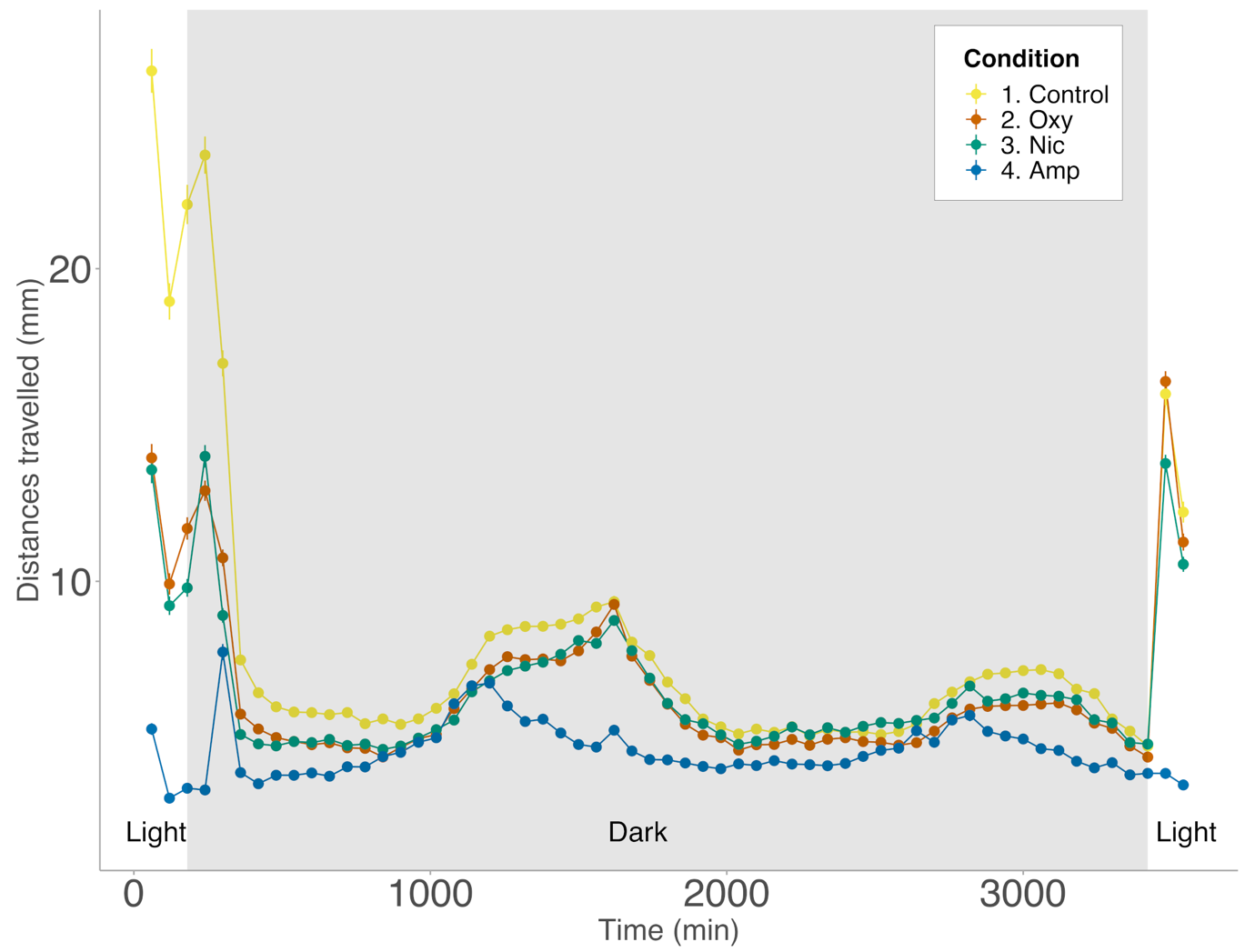


**Supplementary Figure 4. Drug exposures do not alter the free running period.** Mean distance travelled per hour by larvae from each treatment group during a prolonged period of darkness (grey). Sample size n=48 for each group. Data shows mean ± SEM.


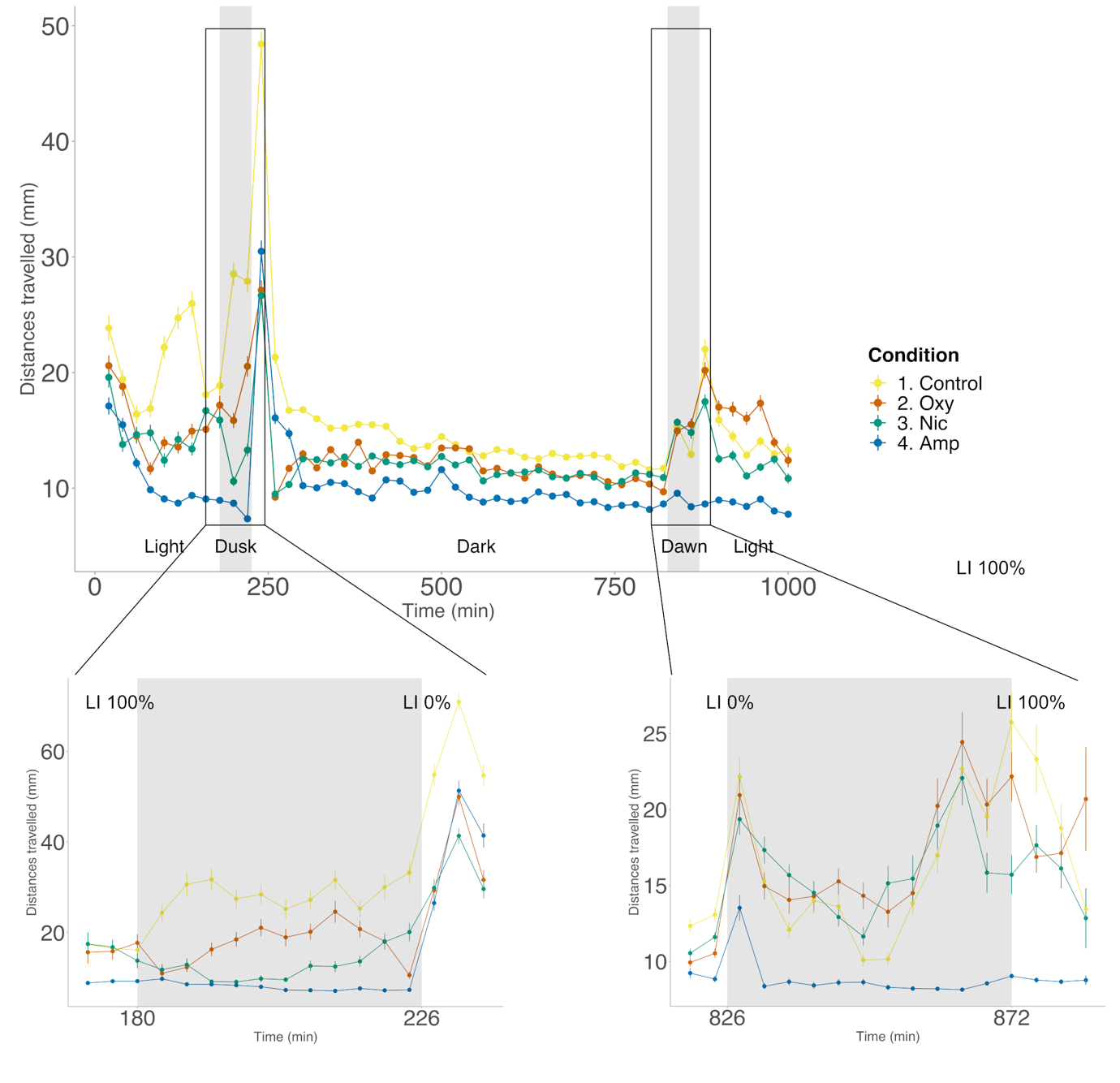


**Supplementary Figure 5. Responses to dusk and dawn following exposure.** Mean distance travelled per 20 minutes by larvae from each treatment group during alternating light and dark conditions with gradual dusk and dawn periods (grey). Response to gradual dusk and dawn is displayed in a 4-min resolution plot. Sample size n=48 for each group. Data shows mean ± SEM.
